# Phosphodiesterase 1C integrates store-operated calcium entry and cAMP signaling in leading-edge protrusions of migrating human arterial myocytes

**DOI:** 10.1016/j.jbc.2021.100606

**Published:** 2021-03-28

**Authors:** Paulina Brzezinska, Nicholas J. Simpson, Fabien Hubert, Ariana N. Jacobs, M. Bibiana Umana, Jodi L. MacKeil, Jonah Burke-Kleinman, Darrin M. Payne, Alastair V. Ferguson, Donald H. Maurice

**Affiliations:** 1 Department of Biomedical and Molecular Sciences, Queen’s University, Kingston, Ontario, Canada; 2 Department of Surgery, Queen’s University, Kingston, Ontario, Canada

**Keywords:** adenylate cyclase, cyclic AMP, calcium, store-operated calcium entry, phosphodiesterases, phosphodiesterase 1C, human arterial smooth muscle cells, ADCY, adenylyl cyclase, BSA, bovine serum albumin, Ca2+, calcium, cAMP, cyclic AMP, CPA, cyclopiazonic acid, DAPI, 4′,6-diamidino-2-phenylindole, FRET, fluorescence resonance energy transfer, fsk, forskolin, HASMC, human arterial smooth muscle cell, HBSS, Hank's balanced salt solution, LEP, leading-edge protrusion, PDE, phosphodiesterase, PDE1C, phosphodiesterase 1C, PKA, protein kinase A, ROI, region of interest, SBMB, smooth muscle basal media, SOCE, store-operated calcium entry, STIM1, stromal interaction molecule 1

## Abstract

In addition to maintaining cellular ER Ca^2+^ stores, store-operated Ca^2+^ entry (SOCE) regulates several Ca^2+^-sensitive cellular enzymes, including certain adenylyl cyclases (ADCYs), enzymes that synthesize the secondary messenger cyclic AMP (cAMP). Ca^2+^, acting with calmodulin, can also increase the activity of PDE1-family phosphodiesterases (PDEs), which cleave the phosphodiester bond of cAMP. Surprisingly, SOCE-regulated cAMP signaling has not been studied in cells expressing both Ca^2+^-sensitive enzymes. Here, we report that depletion of ER Ca^2+^ activates PDE1C in human arterial smooth muscle cells (HASMCs). Inhibiting the activation of PDE1C reduced the magnitude of both SOCE and subsequent Ca^2+^/calmodulin–mediated activation of ADCY8 in these cells. Because inhibiting or silencing Ca^2+^-insensitive PDEs had no such effects, these data identify PDE1C-mediated hydrolysis of cAMP as a novel and important link between SOCE and its activation of ADCY8. Functionally, we showed that PDE1C regulated the formation of leading-edge protrusions in HASMCs, a critical early event in cell migration. Indeed, we found that PDE1C populated the tips of newly forming leading-edge protrusions in polarized HASMCs, and co-localized with ADCY8, the Ca^2+^ release activated Ca^2+^ channel subunit, Orai1, the cAMP-effector, protein kinase A, and an A-kinase anchoring protein, AKAP79. Because this polarization could allow PDE1C to control cAMP signaling in a hyper-localized manner, we suggest that PDE1C-selective therapeutic agents could offer increased spatial specificity in HASMCs over agents that regulate cAMP globally in cells. Similarly, such agents could also prove useful in regulating crosstalk between Ca^2+^/cAMP signaling in other cells in which dysregulated migration contributes to human pathology, including certain cancers.

Store-operated Ca^2+^ entry (SOCE) allows extracellular Ca^2+^ to replenish depleted endoplasmic reticulum (ER) (Ca^2+^) stores in nonexcitable and certain excitable cells (1, 2, 3). For instance, inositol trisphosphate-mediated Ca^2+^ release at the leading edge of polarized migrating cells can deplete local ER(Ca^2+^) stores and activate SOCE (4, 5). SOCE occurs through Ca^2+^-selective, Ca^2+^ release-activated Orai channels, the best studied of which is Orai1 (1, 2, 3, 6). Orai1 is activated upon its interaction with clusters of an extended form of the resident ER Ca^2+^-sensing protein, stromal interaction molecule 1 (STIM1); STIM1 adopts its extended conformation in response to reduced interactions with depleted ER luminal Ca^2+^ (1, 2, 3). While SOCE dysregulation contributes to immunodeficiencies, neurodegenerative diseases and can promote arterial intimal hyperplasia (1, 2, 3, 6), translational opportunities offered by targeting this system are currently limited.

In addition to its role in maintaining ER(Ca^2+^) stores, SOCE-sourced Ca^2+^ also influences the activity of certain Ca^2+^-sensitive cellular enzymes. For instance, the Ca^2+^-sensitive subgroup of adenylyl cyclases (ADCYs) are uniquely sensitive to SOCE-derived Ca^2+^ (7). Indeed, SOCE increases cAMP synthesis in cells expressing heterologously either ADCY1 or ADCY8, Ca^2+^/ calmodulin (CaM)-activated forms of ADCY (7, 8, 9, 10, 11, 12, 13, 14, 15, 16). At a molecular level, recent studies have shown that SOCE-mediated activation of ADCY8 occurs locally in cells and requires co-localization and physical interactions between several proteins, including Orai1 and ADCY8 (11, 16). Phosphorylation(s) catalyzed by the cAMP effector, protein kinase A (PKA), inactivates Orai1, allowing reciprocal regulation of these signaling systems (16). Although SOCE reduces cAMP synthesis in cells heterologously expressing ADCY5 or ADCY6, Ca^2+^-inhibited forms of ADCY (13), the molecular events underpinning these events remain poorly understood. Interestingly, cAMP levels and PKA activity are also increased in certain cell types in response to ER(Ca^2+^) store depletion, independently of changes in cytosolic Ca^2+^ (8). In this case, plasma membrane translocation of STIM1 seems to be critical, and evidence indicates that another ADCY variant, ADCY3, may be involved (17).

Human arterial smooth muscle cells (HASMCs) in healthy arteries express a “contractile” phenotype. “Contractile” HASMCs are excitable cells with limited proliferative or migratory capacities (18). In response to stressors *in vivo* or exposure to growth factors in culture *in vitro*, HASMCs can adopt an “activated” phenotype (18). “Activated” HASMCs are nonexcitable cells with marked proliferative and migratory capacities (18). Compared with contractile HASMCs, activated HASMCs express elevated levels of the elements required for SOCE (Orai1, STIM1, and inositol trisphosphate receptors) but reduced levels of the voltage-gated Ca^2+^ channels which dominate in contractile HASMCs (19). In addition, these phenotypically distinct HASMCs also differentially express variants of enzymes involved in cAMP-signaling. Thus, while the Ca^2+^-inhibited ADCY6 is dominant in both contractile and activated HASMCs (20), induction of the Ca^2+^/CaM-activated ADCY8 accompanies adoption of the activated HASMC phenotype (21). In addition, contractile and activated HASMCs each express unique combinations of cyclic nucleotide phosphodiesterases (PDEs), the sole intracellular enzymes capable of hydrolyzing and inactivating cAMP or cGMP. HASMCs expressing either contractile or activated phenotypes express a cGMP-hydrolyzing Ca^2+^/CaM-activated PDEs (PDE1B) and numerous Ca^2+^-insensitive cAMP-hydrolyzing PDEs (PDE3, PDE4, and PDE5). In contrast, only phenotypically activated HASMCs express a Ca^2+^/CaM-activated cAMP PDE (*i.e.*, PDE1C) (22, 23, 24, 25, 26, 27, 28, 29). Although PDE1C is readily activated by Ca^2+^/CaM *in vitro*, the intracellular sources of Ca^2+^ that activate this enzyme in cells are less clear.

To date, few studies have comprehensively investigated the impact of SOCE on cAMP-signaling in HASMCs. Further, no studies have assessed how expression of a Ca^2+^/CaM-activated, cAMP-hydrolyzing, PDE (*i.e.*, PDE1C) in cells would influence SOCE-mediated changes in cAMP synthesis in cells expressing Ca^2+^-sensitive ADCYs. Herein, we describe results of experiments aimed at answering each of these important questions. First, we show that depleting ER(Ca^2+^) stores activates PDE1C. Second, we show that inhibiting this PDE1C activation reduces SOCE as well as SOCE-dependent ADCY8 activation in these cells. Functionally, we show that PDE1C-mediated cAMP hydrolysis, through its impact on SOCE-mediated activation of ADCY8 in HASMCs, controls formation of actin-rich leading-edge protrusions (LEPs) in polarized migrating HASMCs. Taken together, our findings identify a novel signaling system through which it may be possible to promote the healing functions associated with HASMC migration and to limit the maladaptive effects of exaggerated HASMC migration in vascular conditions, including atherosclerosis or poststenting restenosis.

## Results

### Impact of ER(Ca^2+^) store depletion or SOCE on HASMC cAMP

**Figure 1:**
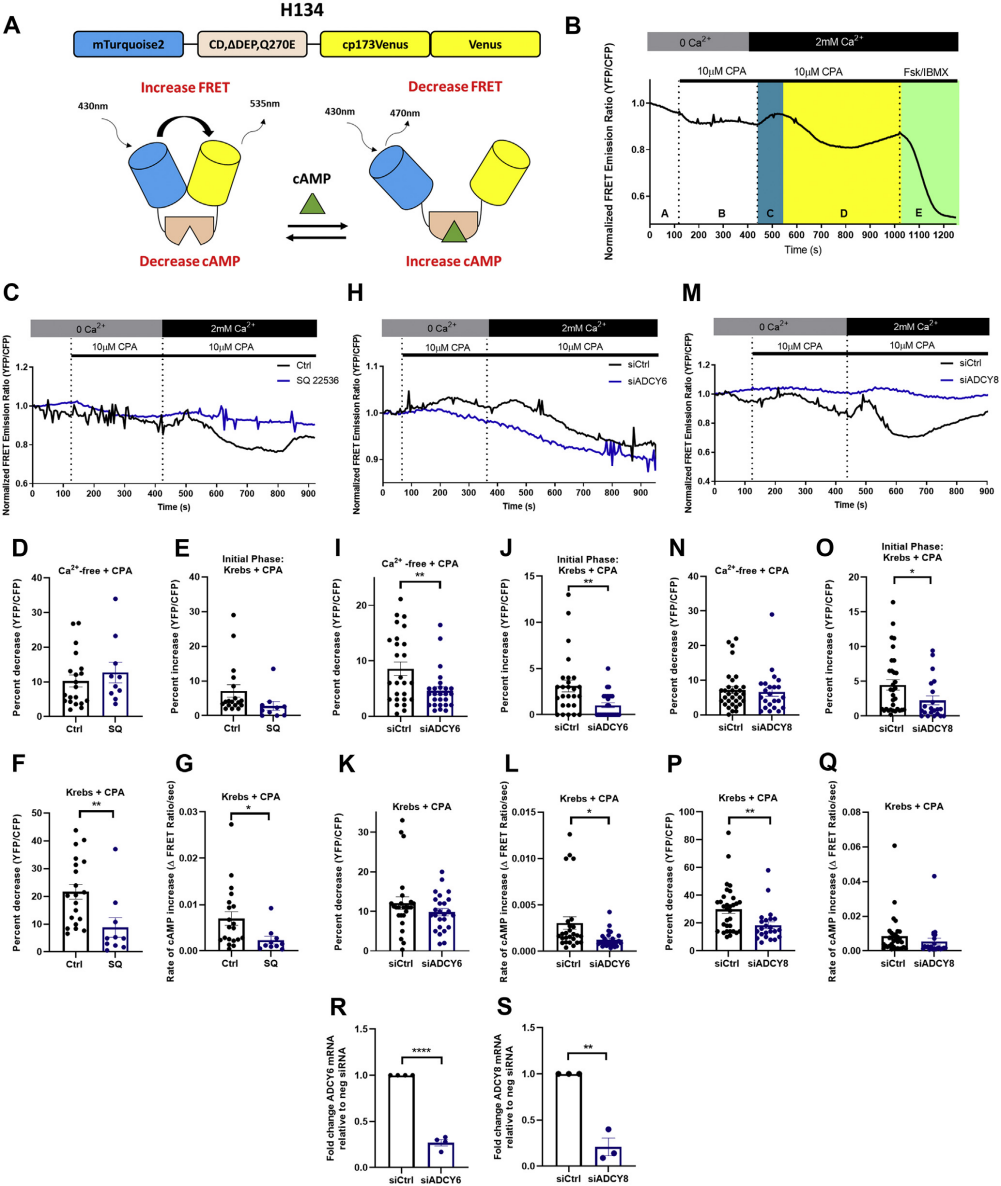
**SOCE-dependent effects on HASMC cAMP.***A*, model of the FRET cAMP sensor, mTurq2ΔEPACcp173Ven_Ven, showing the EPAC1 cAMP-binding domain (CD, ΔDEP, Q270E) positioned between a mTurquoise2 (donor domain, *blue*) and two Venus (acceptor, *yellow*) domains (*top*), and representing how cAMP binding increases the distance between the donor and acceptor domains, thus reducing FRET (*bottom*). *B*, representative trace of normalized FRET emission ratio measured in H134-expressing HASMCs under initial experimental conditions (Ca^2+^-free Krebs buffer, “A”) during ER(Ca^2+^) store depletion (Ca^2+^-free Krebs buffer supplemented with CPA [10 μM], “B”), during the early (*blue*, “C”) and late (*yellow*, “D”) phases of SOCE [Krebs buffer supplemented with CPA (10 μM)] and, lastly, during sensor saturation [Forskolin (10 μM) + IBMX (100 μM), Fsk/IBMX, “E”]. *C*, representative single cell traces of normalized FRET emission ratios measured in H134-expressing HASMCs treated as in *B* in the presence of either saline (*black*) or of SQ 22536 (1 mM). *D*–*G*, changes in normalized FRET emission ratios measured in control (n = 20) or SQ22536-treated (n = 10) HASMCs during ER(Ca^2+^) store depletions (*D*), the early phase of SOCE (*E*), or the late phase (*F* and *G*) (Student’s unpaired *t* test, ∗*p* = 0.0420 ∗∗*p* = 0.0083). *H*, representative single-cell traces of normalized FRET emission ratios measured in control (siCtrl) or ADCY6-silenced (siADCY6) HASMCs treated as in *B*. *I*–*L*, differences in normalized FRET emission ratios measured in control (n = 26) or ADCY6-silenced (n = 26) HASMCs during ER(Ca^2+^) store depletion (*I*), the early phase of SOCE (*J*), or the late phase (*K* and *L*) of SOCE, respectively (Student’s unpaired *t* test, ∗∗*p* = 0.0076 comparing cAMP increase in Ca^2+^ free Krebs with CPA, ∗∗*p* = 0.004 comparing cAMP decrease during initial phase of SOCE, ∗*p* = 0.01 comparing cAMP rate of increase during SOCE). *M*, representative single-cell traces of normalized FRET emission ratios measured in control (siCtrl) or ADCY8-silenced (siADCY8) HASMCs treated as in *B*. *N*–*Q*, differences in normalized FRET emission ratios measured in control (n = 32) or ADCY8-silenced (n = 22) HASMCs during ER(Ca^2+^) store depletions (*N*), during the early phase of SOCE (*O*) or the late phase (*P* and *Q*) of SOCE, respectively (Student’s unpaired *t* test, ∗*p* = 0.04, ∗∗*p* = 0.007). *R*, levels of ADCY6 mRNA in siCtrl and siADCY6 transfected HASMCs (Student’s unpaired *t* test, ∗∗∗∗*p* < 0.0001). *S*, levels of ADCY8 mRNA in siCtrl and siADCY8 transfected HASMCs (Student’s unpaired *t* test, ∗∗*p* = 0.0012). ADCY, adenylyl cyclase; cAMP, cyclic AMP; CPA, cyclopiazonic acid; EPAC1, exchange protein activated by cAMP-1; FRET, fluorescence resonance energy transfer; HASMC, human arterial smooth muscle cell; SOCE, store-operated calcium entry.

HASMC cAMP levels were monitored in cells expressing a validated cAMP sensor (mTurq2ΔEPACcp173Ven_Ven; (H134)) (30) (Fig. 1*A*). As predicted, decreases in fluorescence resonance energy transfer (FRET) (*i.e.*, increases in cAMP) were recorded in H134-expressing HASMCs incubated with the ADCY activator forskolin (Fsk), and these responses were blocked by the transmembrane ADCY inhibitor, SQ22536 (Fig. S1, *A* and *B*). ER(Ca^2+^) store depletion (Ca^2+^-free Krebs buffer + cyclopiazonic acid (CPA, 10 μM) also decreased FRET (*i.e.*, increased cAMP) (area “B” in representative trace, Fig. 1*B*) in H134-expressing HASMCs. Indeed, in the 139 cells studied, ER(Ca^2+^) store depletion increased cAMP by an average of 8.3 ± 0.7% (mean ± SEM). Subsequent SOCE (Krebs + CPA) caused temporally biphasic changes in cAMP in these H134-expressing HASMCs (areas “C” and “D” in representative trace, Fig. 1*B*). SOCE was associated with an initial transient decrease in cAMP (5.2 ± 0.7%, mean ± SEM) which was followed by larger and longer-lived increase in cAMP (22.1 ± 1.2%, mean ± SEM). Of note, SOCE-associated increases in HASMC cAMP were on average 46.5 ± 1.4% (mean ± SEM) of the maximum increase recorded in these cells [Fsk (10 μM) + 3-isobutyl-1-methylxanthine (IBMX, 100 μM)] (area “E”, representative trace, Fig. 1*B*).

Transmembrane ADCY inhibition with SQ22536 did not alter cAMP changes associated with ER(Ca^2+^) store depletion [Fig. 1*C* (representative tracings), Fig. 1*D* (quantitation)]. While the initial transient decrease in cAMP associated with SOCE was reduced somewhat by SQ22536, this did not reach significance (Fig. 1, *C* and *E*). In contrast, SQ22536 did reduce markedly the magnitude and rate of the increase in cAMP observed later during SOCE (Fig. 1, *C*, *F* and *G*). To determine whether these effects reflected Ca^2+^-mediated effects on the activities of the Ca^2+^-sensitive ADCYs expressed in HASMCs (*i.e.*, ADCY6 and ADCY8), experiments were also conducted in ADCY6-silenced or ADCY8-silenced HASMCs, respectively. Silencing ADCY6, but not ADCY8, blunted cAMP increases associated with ER(Ca^2+^) store depletion (compare Fig. 1, *H* and *I* and Fig. 1, *M* and *N*). Similarly, SOCE-associated changes in cAMP were also differentially affected by silencing of ADCY6 or ADCY8. Indeed, while silencing either of these ADCYs did significantly reduce the magnitude of the early SOCE-associated decrease in cAMP (compare Fig. 1*J* and Fig. 1*O*), only silencing ADCY8 significantly reduced the subsequent SOCE-associated increase in cAMP (compare Fig. 1*K* and Fig. 1*P*). Interestingly, although ADCY6 did not reduce the magnitude of the SOCE-associated increase in cAMP, it did reduce the rate at which cAMP accumulated during this phase of the response (Fig. 1*L*). Because the silencing efficiency of the ADCYs were similar (Fig. 1, *R* and *S*), we suggest this did not represent an important factor in the different responses seen in these experiments. Taken together, our data show that ADCY6 activity was involved in regulating changes in HASMC cAMP observed during ER(Ca^2+^) store depletion and early during SOCE and that ADCY8 activity was dominant in regulating the cAMP increases triggered by SOCE in these cells.

Previous reports indicated that SOCE-mediated activation of ADCY8 could increase cellular PKA activity (16, 31). To determine if HASMC PKA was activated during ER(Ca^2+^) store depletion or in response to SOCE, we monitored PKA activity in cells expressing a PKA activity sensor (AKAR4 FRET sensor (32)) (Fig. S2, *A* and *B*). In these experiments, we observed that PKA activity was elevated during both ER(Ca^2+^) store depletion and SOCE (Fig. S2, *B* and *C*), with a larger fractional increase being associated with SOCE (Fig. S2, *D* and *E*).

### PDE1C regulates SOCE

**Figure 2:**
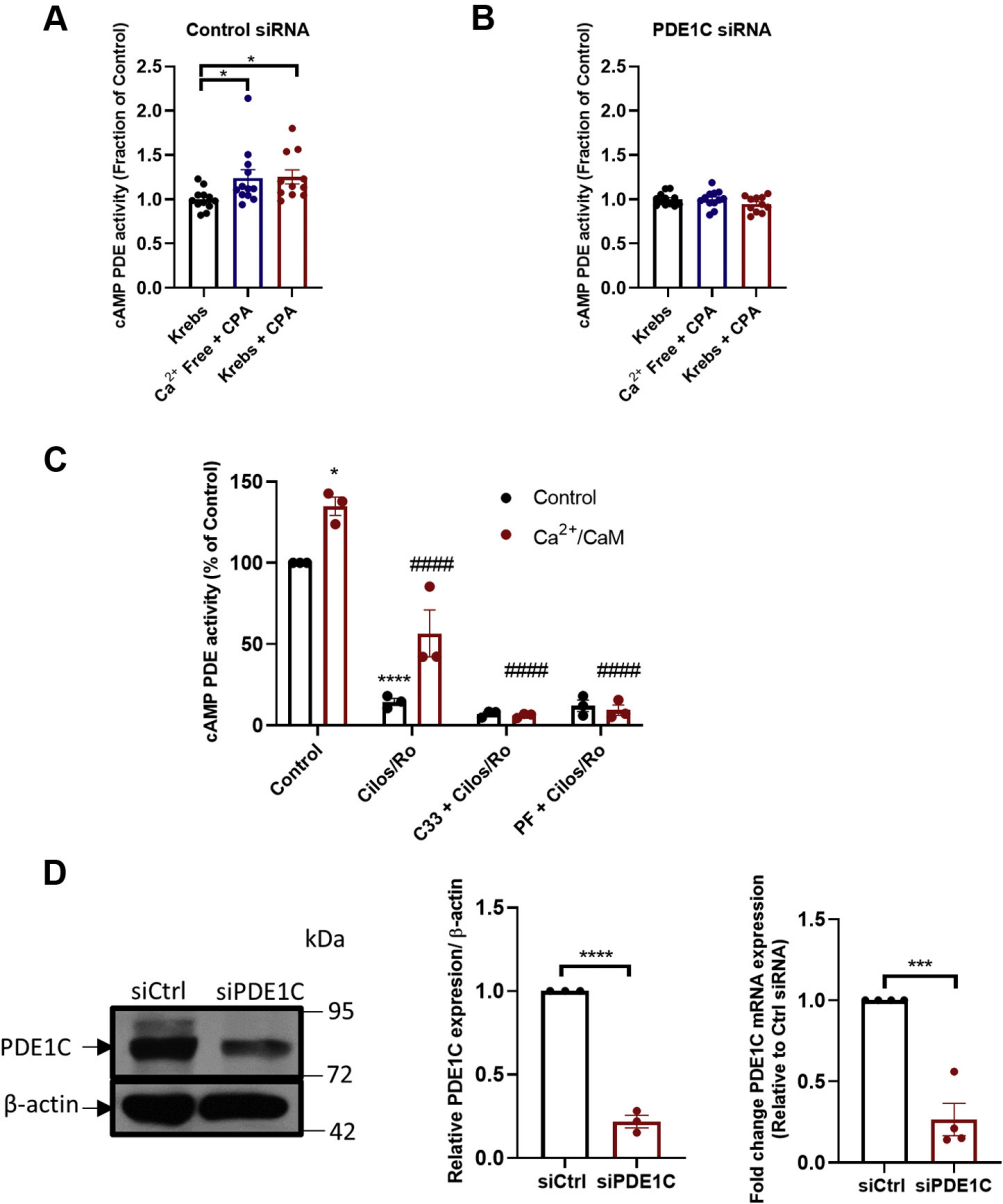
**HASMC PDE1C activation in response to ER (Ca**^**2+**^**) store depletion or SOCE activation.***A* and *B*, cAMP PDE activity in homogenates generated from control siRNA (*A*) or PDE1C siRNA (*B*), transfected HASMCs under control conditions (Krebs), following ER(Ca^2+^) store depletion [Ca^2+^ free + CPA (10 μM)] or following stepwise ER(Ca^2+^) depletion and SOCE activation [Krebs + CPA (10 μM)]. n = 4 experiments where each experiment was carried out in triplicate. One-way ANOVA, F = 3.76, *p* = 0.0343, Dunnett’s multiple comparison’s test was conducted, ∗*p* = 0.0485 control cells (Krebs) *versus* either ER(Ca^2+^) depletion and ∗*p* = 0.0415 control cells (Krebs) *versus* SOCE activation. *C*, HASMC cAMP PDE activity in homogenates without (*black*) or with Ca^2+^/CaM (*red*) in the presence of a combination of cilostamide (5 μM) + Ro 20 to 1724 (10 μM), a combination of C33 (1 μM) and cilostamide (5 μM) + Ro 20 to 1724 (10 μM), or a combination of PF-04827736 (1 μM) and cilostamide (5 μM) + Ro-20 to 1724 (10 μM). n = 3 experiments where each experiment was carried out in triplicate. Tukey’s multiple comparisons test was conducted, ∗*p* = 0.011 compared with control, ∗∗∗∗*p* < 0.0001 compared with control, ####*p* < 0.0001 compared with Ca^2+^/CaM. Two-way ANOVA, F (3, 16) = 8.207, *p* = 0.0016 interaction between control *versus* Ca^2+^/CaM conditions, F (3, 16) = 157.9 within each individual drug condition (control *versus* Ca^2+^/CaM treatment), *p* < 0.0001, F (1, 16) = 19.96, *p* = 0.0004 between the different drug treatments. Tukey’s multiple comparisons test was conducted, ∗*p* = 0.011 compared with control, ∗∗∗∗*p* < 0.0001 compared with control, ####*p* < 0.0001 compared with Ca^2+^/CaM. *D*, representative anti-PDE1C immunoblot of lysates generated from control siRNA-transfected HASMCs (siCtrl) or PDE1C-silenced (siPDE1C) HASMCs and quantitation of the efficiency of PDE1C-silencing in these cells (plot). ∗∗∗*p* < 0.01 *versus* siCtrl, n = 3, ∗∗∗∗*p* < 0.001 *versus* siCtrl, n = 3, Student’s unpaired *t* test. CaM, calmodulin; cAMP, cyclic AMP; CPA, cyclopiazonic acid; HASMC, human arterial smooth muscle cell; PDE, phosphodiesterase; PDE1C, phosphodiesterase 1C; SOCE, store-operated calcium entry.

**Figure 3:**
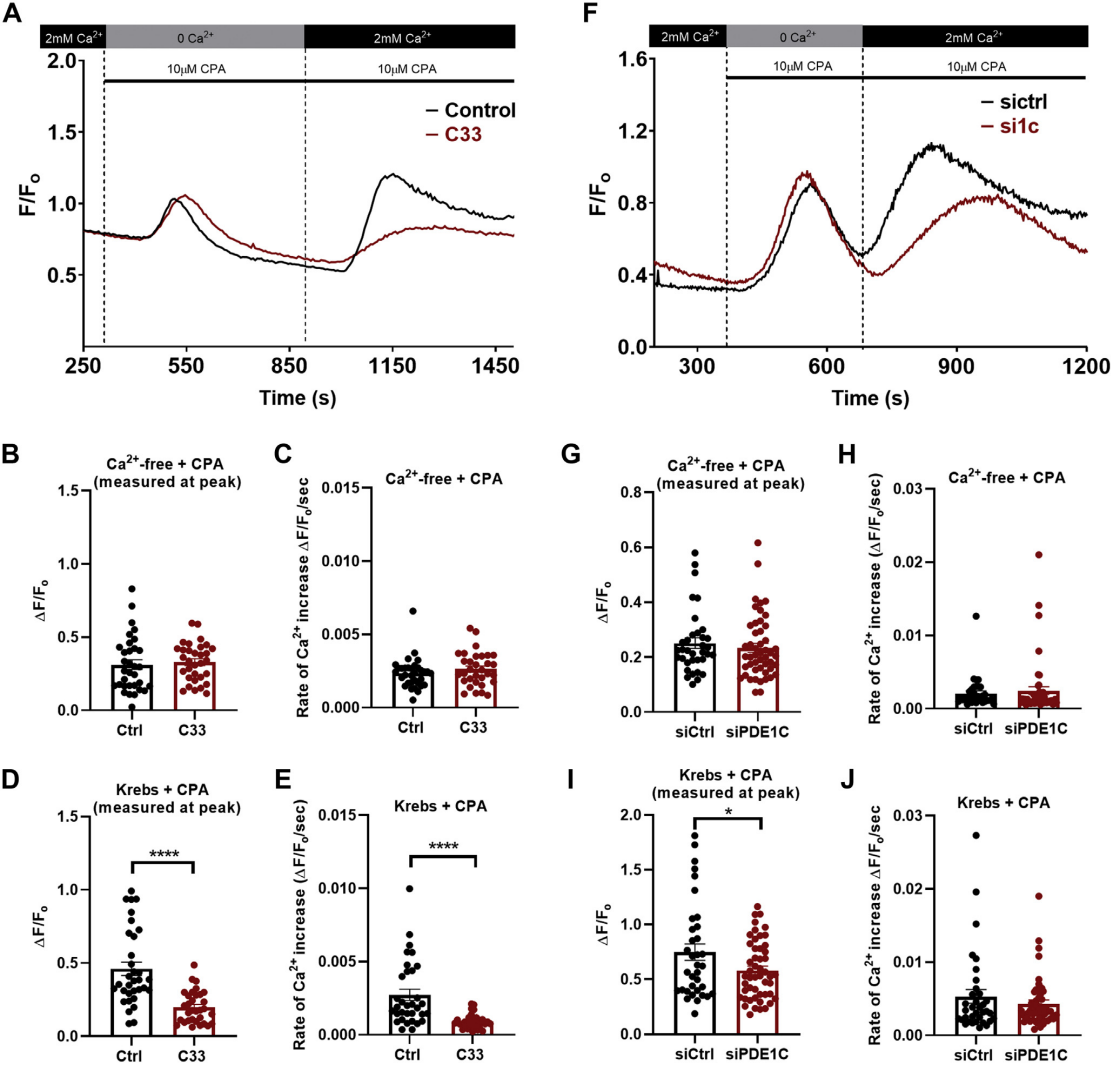
**PDE1C inhibition or PDE1C silencing reduces SOCE in HASMCs.***A*, representative single-cell traces of changes in the fluorescence ratio (F/Fo) of fura-2 in control [vehicle, (DMSO, 0.01% v/v), *black line*] or C33 (1 μM)-treated (*red line*) HASMCs during ER(Ca^2+^) store depletion [0 Ca^2+^ + CPA (10 μM)] or during subsequent SOCE [2 mM Ca^2+^ + CPA (10 μM)]. *B*–*E*, impact of C33 on maximal [Ca^2+^] increases (*B*) and rate of increase (*C*) during ER(Ca^2+^) store depletions or maximal [Ca^2+^] increases (*D*) and rate of increase (*E*) during SOCE. Control n = 33, C33 n = 32 individual cells in which each treatment was analyzed. Student’s unpaired *t* test, ∗∗∗∗*p* < 0.0001 control *versus* C33 treated cells. *F*, representative single-cell traces of changes in the fluorescence ratio (F/Fo) of fura-2 in control siRNA transfected (*black* line) or PDE1C siRNA-transfected (*red line*) HASMCs during ER(Ca^2+^) store depletion [0 Ca^2+^ + CPA (10 μM)] or during subsequent SOCE [2 mM Ca^2+^ +CPA (10 μM)]. *G*–*J*, Impact of PDE1C-silencing on maximal [Ca^2+^] increases (*G*) and rate of increase (*H*) during ER(Ca^2+^) store depletions or maximal [Ca^2+^] increases (*I*) and rate of increase (*J*) during SOCE. siCtrl n = 35, siPDE1C n = 49 individual cells in which treatment was analyzed. Student’s unpaired *t* test, ∗*p* = 0.0340 siCtrl *versus* siPDE1C transfected cells. CPA, cyclopiazonic acid; HASMC, human arterial smooth muscle cell; PDE, phosphodiesterase; PDE1C, phosphodiesterase 1C; SOCE, store-operated calcium entry.

ER(Ca^2+^) store depletion and SOCE each increased cAMP PDE activity in HASMCs (Fig. 2*A*) and silencing the Ca^2+^-sensitive cAMP PDE (*i.e.*, PDE1C) obviated these effects (Fig. 2, *B* and *D*). Because silencing PDE1C did not alter Ca^2+^-insensitive cAMP PDE activity levels in these cells (Table S4), these data indicated that HASMC PDE1C was activated in response to these treatments. To investigate whether PDE1C activation reciprocally regulated ER(Ca^2+^) store depletion, or SOCE, we next studied how its inhibition or silencing altered their associated intracellular Ca^2+^ transients. For these experiments, PDE1C was inhibited using either one of two structurally distinct PDE1-family selective inhibitors, C33 or PF-04827736 (Fig. S3, Fig. 2*C*) [34–35], and PDE1C was silenced with one of two selective PDE1C-targetting siRNAs (Fig. 2*D* and Fig. S7). PDE1C inhibition did not alter the maximum increase or rate of rise in intracellular Ca^2+^ during ER(Ca^2+^) store depletion (Fig. 3*A* (representative traces), Fig. 3, *B* and *C* (quantitation)). In contrast, PDE1C inhibition did reduce the maximum level of Ca^2+^ entry detected during SOCE and its rate of entry in HASMCs (Fig. 3, *A*, *D* and *E*). Consistent with the idea that PDE1 inhibitors acted specifically on PDE1C, silencing this PDE significantly reduced SOCE in these cells [Fig. 3*F* (representative traces), Fig. 3, *G*–*J* (quantitation)]. Because inhibition or silencing of the dominant Ca^2+^-insensitive PDEs only modestly altered maximal Ca^2+^ increases during SOCE in HASMCs (Fig. S4, *A*–*C*), our data identify PDE1C as a selective regulator of cAMP’s actions on SOCE in these cells.

Orai1/STIM1-containing microscopic plasma membrane *puncta* form before SOCE in most nonexcitable cells (33, 34). While our analysis confirmed that ER(Ca^2+^) store depletion did promote formation of Orai-1/Stim-1-containing *puncta* in HASMCs, silencing PDE1C did not alter their numbers or the expression of the ER luminal Ca^2+^-sensing protein, Stim-1 (Fig. S5, *A*–*D*). These findings indicate that PDE1C is unlikely to regulate HASMC SOCE by altering ER(Ca^2+^) sensing or the dynamics of Orai1-Stim-1 interactions in these cells.

### PDE1C activation promotes SOCE-mediated ADCY8 activation

**Figure 4:**
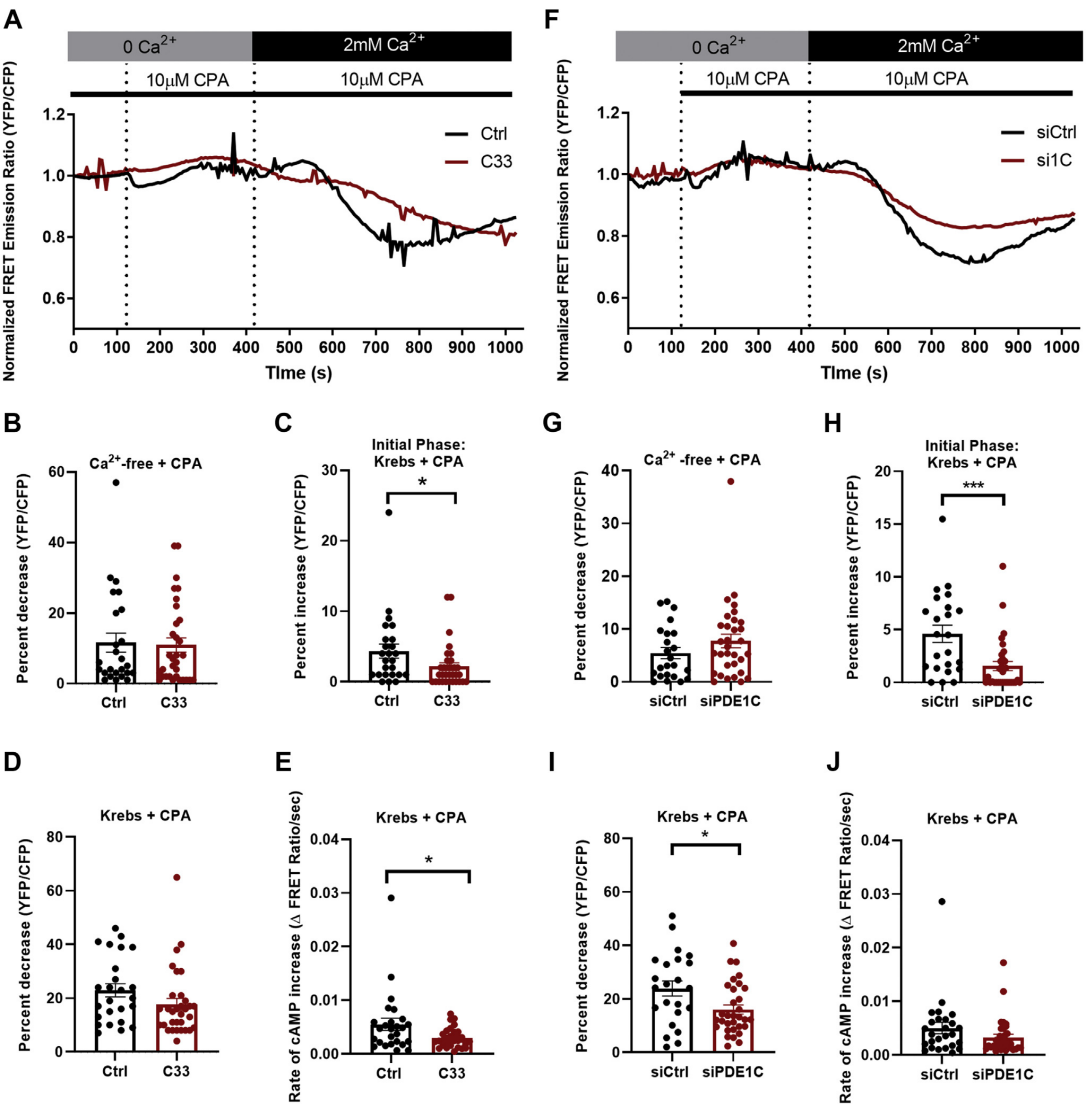
**PDE1C inhibition or PDE1C silencing, inhibit, rather than promote, SOCE-associated increases in HASMCs cAMP.***A*, representative single cell traces of normalized FRET emission ratios measured in H134-expressing HASMCs treated as in Figure 1*B* in the absence (*black line*) or the presence (*red line*) of C33 (1 μM). *B*–*D*, changes in normalized FRET emission ratios measured in control (n = 25) or C33-treated (n = 32) HASMCs during (*B*) ER(Ca^2+^) store depletions, (*C*) the early phase of SOCE or (*D*) the later phase of SOCE. *E*, rate of change in cAMP during the later phase of SOCE in HASMC; ∗*p* < 0.05, Student’s unpaired *t* test. *F*, representative single cell traces of normalized FRET emission ratios measured in control siRNA-transfected H134-expressing HASMCs (*black line*) or PDE1C-silenced H134-expressing HASMCs treated as in Figure 1*B*. *G*–*I*, changes in normalized FRET emission ratios measured in control (n = 23) or PDE1C-silenced (n = 32) HASMCs during (*G*) ER(Ca^2+^) store depletion, (*H*) the early phase of SOCE or (*I*) the later phase of SOCE. *J*, rate of change in cAMP during the later phase of SOCE in HASMCs. ∗*p* < 0.05, ∗∗∗*p* < 0.001, Student’s unpaired *t* test. cAMP, cyclic AMP; FRET, fluorescence resonance energy transfer; HASMC, human arterial smooth muscle cell; PDE, phosphodiesterase; PDE1C, phosphodiesterase 1C; SOCE, store-operated calcium entry.

PDE1C inhibition did not alter basal HASMC cAMP (0 Ca^2+^) nor the increase caused by ER(Ca^2+^) store depletion (0 Ca^2+^ + CPA) (Fig. 4*A* (representative traces), Fig. 4*B* (quantitation)). In contrast, inhibiting PDE1C reduced the early SOCE-associated decrease in cAMP (Fig. 4, *A* and *C*) and the rate at which cAMP accumulated during the latter phase of SOCE (Fig. 4, *A*, *D* and *E*). Similarly, silencing PDE1C did not alter cAMP increases caused by HASMC ER(Ca^2+^) store depletion (Fig. 4, *F* and *G*) but did reduce the magnitude of both the early and late SOCE-associated changes in cAMP (Fig. 4, *F* and *H*–*J*). Interestingly and consistent with the idea that PDE1C is unique in its ability to affect SOCE-associated changes in HASMC cAMP, although PDE4 inhibition (Ro, 20–1724, 10 μM) significantly increased basal cAMP in H134-expressing HASMCs (15.9 ± 1.9%, n = 20 cells), the temporally biphasic changes in cAMP associated with SOCE activation were completely unaltered by inclusion of the PDE4 inhibitor in our experiments (Fig. S6, *A*–*D*). Taken together, these data support the hypothesis that PDE1C activation, perhaps in combination with ADCY6 inhibition, contributes to the early transient decrease in cAMP seen during SOCE. Moreover, they support the idea that inhibiting, or silencing, PDE1C, perhaps by reducing SOCE, decreases SOCE-mediated activation of ADCY8 in these cells. Lastly, given that PDE4 activity accounts for ∼65% of HASMC cAMP PDE activity (Table S4), these data also support the notion that PDE1C and PDE4s regulate distinct HASMC cAMP “pools” and that the cAMP pool regulated by PDE1C may “gate” SOCE-mediated ADCY8 activation.

### PDE1C regulates formation of leading-edge protruding structures in polarized HASMCs

Recently, we reported that inhibiting or silencing PDE1C increased leading-edge protruding structure formation in polarized motile HASMCs (35). Interestingly, similarly targeting Ca^2+^-insensitive PDEs (*i.e.*, PDE3, PDE4) in these cells reduced, rather than promoted, formation of these structures (35). Because the data presented here indicated that PDE1C activation was necessary for optimal SOCE-dependent ADCY8 activation (Fig. 4), we investigated whether PDE1C regulated HASMC LEP formation by virtue of its effects on ADCY8 activity. Moreover, because SOCE effectively modulated cAMP levels even in cells in which the dominant Ca^2+^-insensitive cAMP PDE activity (PDE4) was inhibited (Fig. S6), we further tested the hypothesis that these events were coordinated locally, perhaps at the front, of polarized HASMCs.

**Figure 5:**
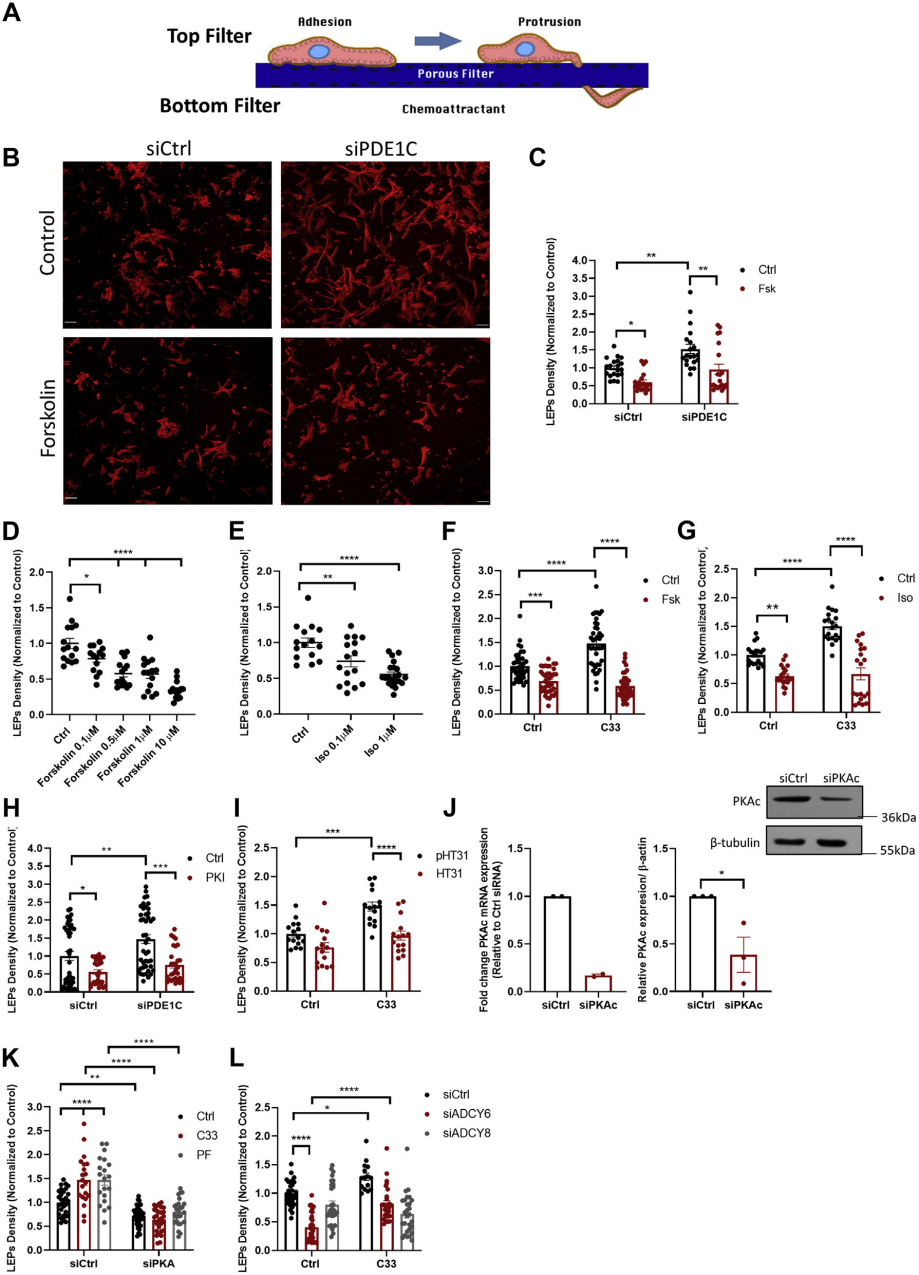
**PDE1C regulates formation of leading-edge protrusions (LEPs) in polarized HASMCs.***A*, model of the assay used to identify and quantify LEPs which accumulate on the underside of FluoroBlok membranes. *B*, representative images showing increased numbers of LEPs (tetramethylrhodamine-isothiocyanate-conjugated phalloidin-stained actin, *red*) formed by PDE1C-silenced HASMCs, compared with controls and inhibition of this process by addition of Forskolin (0.5 μM), scale bars 50 μm. *C*, LEP quantitation. LEP abundance in multiple individual membranes was determined by averaging phalloidin fluorescence in 4 to 5 separate nonoverlapping areas on individual membranes. n = 4 experiments in which individual experiments were carried out with four separate FluoroBlok membranes. A two-way ANOVA followed by Tukey’s multiple comparisons was conducted, ∗*p* < 0.05, ∗∗*p* < 0.01 between experimental conditions. *D* and *E*, concentration-dependent inhibition of LEP formation in HASMCs incubated with forskolin (Fsk) or isoproterenol (Iso). n = 3 experiments. A two-way ANOVA followed by Tukey’s multiple comparisons was conducted; 1-way ANOVA: Fsk dose response: F = 21.77, *p* < 0.0001. Dunnett’s multiple comparisons: ∗*p* = 0.0222 control *versus* Fsk 0.1 μM, ∗∗∗∗*p* < 0.0001 control *versus* Fsk (0.5 μM, 1 μM, 10 μM). 1-way ANOVA: Iso dose response: F = 14.85, *p* < 0.0001. Dunnett’s multiple comparisons: ∗*p* = 0.0080 control *versus* Iso 0.1 μM, ∗∗∗∗*p* < 0.0001 control *versus* Iso 1 μM. *F*–*I*, inhibition of LEP formation in HASMCs incubated with (*F*, *G,I* and *L*) the PDE1-family inhibitor C33 (1 μM) or in which cells were transfected with siPDE1C (*H*) in the absence or presence of 0.5 μM Fsk (*F*), 1 μM Iso (*G*), 40 μM myrPKI (*H*), or 10 μM HT31, or an inactive peptide (40 μM pHT31) (*I*). n = 3 to 5 experiments. Two-way ANOVA: C33 + siPDE1C: F (1, 90) = 7.486, *p* = 0.0075 interaction, F (1, 90) = 6.120, *p* = 0.0152 (control *versus* C33), F (1, 90) = 14.78, *p* = 0.0002 (siCtrl *versus* siPDE1C transfected cells). Two-way ANOVA: PDE1C Fsk: F (1, 76) = 0.6591, *p* = 0.4194 interaction, F (1, 76) = 17.32, *p* < 0.0001 siCtrl *versus* siPDE1C, F (1, 76) = 20.71, *p* < 0.0001 control *versus* forskolin treated cells. Tukey’s multiple comparisons: ∗*p* = 0.0479, ∗∗*p* = 0.0040 (siCtrl *versus* siPDE1C control), ∗∗*p* = 0.0017 (siPDE1C control *versus* siPDE1C + forskolin). Two-way ANOVA: C33 HT31: F (1, 56) = 3.201, *p* = 0.0790 interaction, F (1, 56) = 20.21, *p* < 0.0001 (control *versus* C33 treated cells), F (1, 56) = 23.97, *p* < 0.0001 (st-HT31(p) *versus* st-HT31 treated cells). Tukey’s multiple comparisons: ∗∗∗*p* = 0.0002 (st-HT31(p) control *versus* st-HT31(p) + C33), ∗∗∗∗*p* < 0.0001 (st-HT31(p) + C33 *versus* st-HT31 + C33). Two-way ANOVA: C33 Iso. F (1, 76) = 12.13, *p* = 0.0008 interaction, F (1, 76) = 16.60, *p* = 0.0001 (control *versus* C33), F (1, 76) = 81.78, *p* < 0.0001 (control *versus* isoproterenol treatment). Tukey’s multiple comparisons: ∗∗*p* = 0.0010 (control *versus* Iso), ∗∗∗∗*p* < 0.0001 (control *versus* C33, and C33 *versus* C33 + Iso). Two-way ANOVA: C33 Fsk: F (1, 155) = 30.00, *p* < 0.0001 interaction, F (1, 155) = 12.18, *p* = 0.0006 (control *versus* C33 treated cells), F (1, 155) = 129.5, *p* < 0.0001 (control *versus* Fsk treated cells). Tukey’s multiple comparisons: ∗∗∗*p* = 0.0003 (control *versus* Fsk), ∗∗∗∗*p* < 0.0001 (control *versus* C33 and C33 *versus* C33 + Fsk). Two-way ANOVA: PKI: F (1, 143) = 1.466, *p* = 0.2280 interaction, F (1, 143) = 8.173, *p* = 0.0049 (sictrl *versus* siPDE1C transfected cells), F (1, 143) = 25.46, *p* < 0.0001 (control *versus* forskolin treated cells). Tukey’s multiple comparisons: ∗*p* = 0.0367 (siCtrl control *versus* PKI), ∗∗*p* = 0.0080 (siCtrl *versus* siPDE1C), ∗∗∗*p* = 0.0001 (siPDE1C control *versus* siPDE1C + PKI). *J* and *K*, silencing HASMC PKA(Cα) (*I*) inhibited basal HASMC LEP formation and antagonized C33 (1 μM) or PF-04827736 (1 μM)-induced increases in LEP formation; data normalized to siRNA control, n = 3 experiments where each used four FluoroBlok membranes. Two-way ANOVA: siPKA C33 and PF: F (2, 155) = 10.84, *p* < 0.0001 interaction, F (1, 155) = 128.1, *p* < 0.0001 (siCtrl *versus* siPKA transfected cells), F (2, 155) = 10.42, *p* < 0.0001 [comparison *versus* drug treatment (control, C33, and PF)]. Tukey’s multiple comparisons: ∗∗*p* < 0.01 (siCtrl *versus* siPKA), ∗∗∗∗*p* < 0.0001 (siCtrl control *versus* each of C33 and PF), (siCtrl + C33 *versus* siPKA + C33) and (siCtrl + PF *versus* siPKA + PF). Two-way ANOVA: siAC8 and siAC6 Fsk: F (2, 154) = 14.65, *p* < 0.0001 interaction, F (1, 154) = 13.63 *p* = 0.0003 (control *versus* C33), F (2, 154) = 40.63, *p* < 0.0001 [comparison of transfection conditions (siCtrl *versus* siADCY6 *versus* siADCY8)]. Tukey’s multiple comparisons test: ∗*p* = 0.0289 (control *versus* C33 treated cells), ∗∗∗∗*p* < 0.0001 (siCtrl *versus* siADCY6 transfected cells and control *versus* C33 in siADCY6 transfected cells). ADCY, adenylyl cyclase; HASMC, human arterial smooth muscle cell; PDE, phosphodiesterase; PDE1C, phosphodiesterase 1C.

Consistent with our previous work (35), silencing PDE1C using either of two PDE1C siRNAs increased LEP formation (Fig. 5, *A*–*C*, Fig. S7, *A*–*C*). In contrast, but as predicted based on previous work (36), ADCY activation with Fsk or the β-adrenergic receptor agonist isoproterenol (Iso) concentration-dependently reduced LEP formation in polarized HASMCs (Fig. 5*B* (representative images), Fig. 5, *C*–*E*) and antagonized the LEP promoting effects of PDE1C inhibition (Fig. 5, *F* and *G*). Consistent with an important role for PKA in these events, addition of the cell-penetrant PKA inhibitory peptide (Myr-PKI) inhibited LEP formation and antagonized the LEP promoting actions of silencing PDE1C (Fig. 5*H*). Addition of a cell-permeant AKAP–PKA disruptor peptide, St-Ht31 (HT31), but not its inactive variant, St-Ht31(P) (pHT31), reversed the LEP promoting effects associated with PDE1C inhibition (Fig. 5*I*), indicating that PKA compartmentation within an AKAP-based signalosome is critical in these events. As was the case with another cAMP effector, exchange protein activated by cAMP-1 (35), silencing HASMC PKA(Cα) (Fig. 5*J*), reduced basal LEP formation and inhibited the LEP promoting effects of each of the two structurally distinct PDE1-family inhibitors used in our studies (Fig. 5*K*). Because our data showed that PDE1C inhibition or silencing of this PDE more markedly impacted ADCY8-associated changes in HASMC cAMP, we next investigated the dependence of this ADCY on LEP formation. Interestingly, although ADCY6-silenced HASMCs produced fewer LEPs than control cells, PDE1C inhibition still promoted LEP formation in these cells (Fig. 5*L*). In marked contrast, while silencing ADCY8 did not statistically reduce LEP formation, PDE1C inhibition did not promote formation of these structures in ADCY8-silenced HASMCs (Fig. 5*L*); an effect consistent with PDE1C acting in concert with ADCY8, but not ADCY6, to regulate LEP formation. Together, these findings show that PDE1C likely acts within an AKAP-based, PKA-containing, and perhaps exchange protein activated by cAMP-1–containing signalosome to regulate HASMC LEP formation and that ADCY8 was dominant in providing the source of cAMP that allowed this system to function in these cells.

Because our data linked SOCE and LEP formation in HASMCs and previous reports indicated that greater SOCE occurred at the front of migrating cells (4, 5, 31), we tested whether SOCE was larger at the leading edge of polarized HASMCs. Consistent with this idea, SOCE was greater at the front than at the cell body in migrating HASMCs [Fig. S8*A* (representative traces), Fig. S8, *B* and *C* (quantitation)].

### Proteins involved in potentiating LEP formation in polarized HASMCs interact and localize preferentially to tips of LEPs

**Figure 6:**
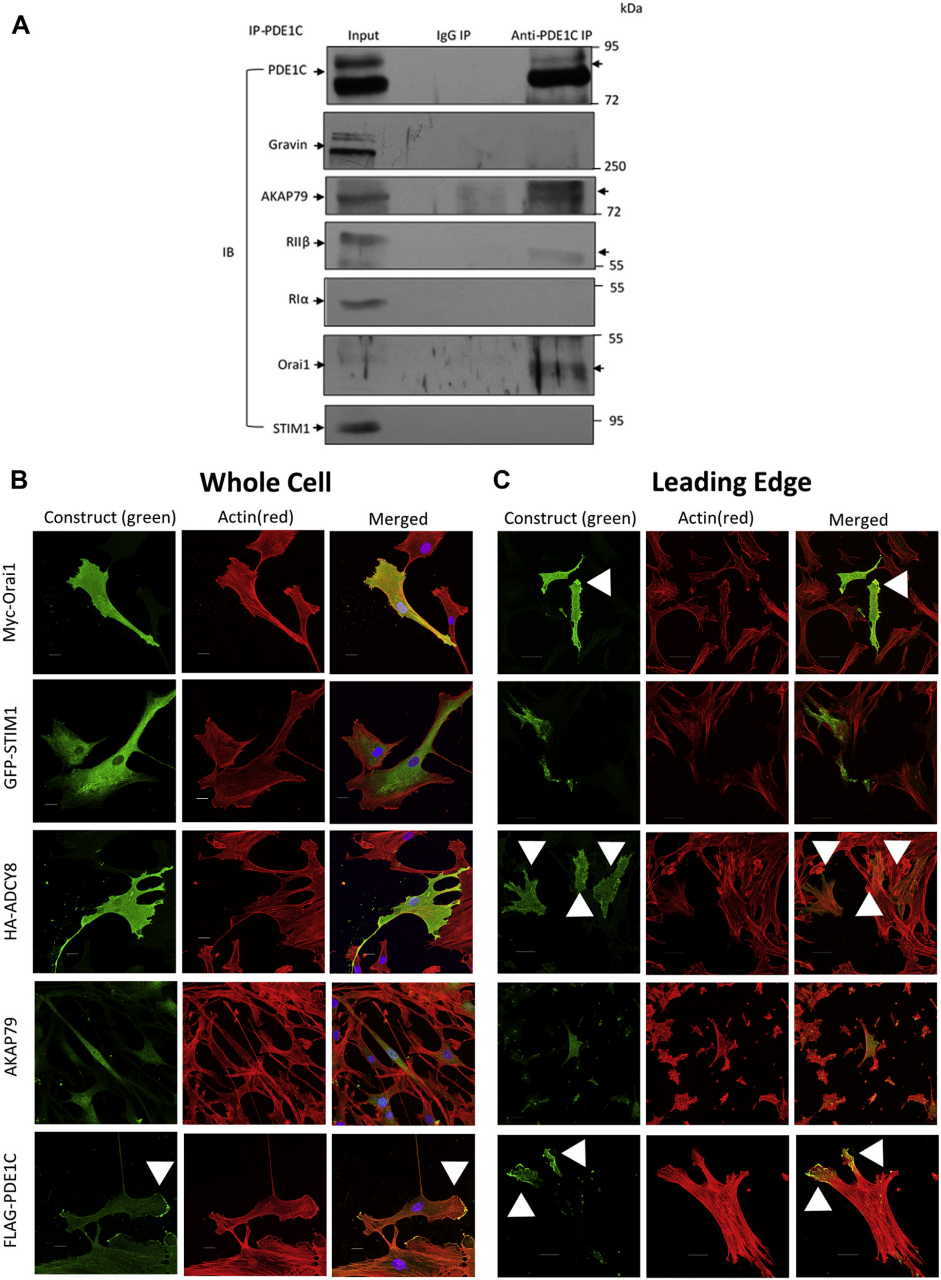
**PDE1C interaction and co-localization with PKA, AKAP79, Orai1, and ADCY8 in HASMC and HASMC LEPs.***A*, representative immunoblots of anti-PDE1C, or anti-IgG, generated immune complexes analyzed for PDE1C, gravin, AKAP79, PKA-RIIβ, PKA-RIα, Orai1, or STIM1. Proteins isolated along with PDE1C in anti-PDE1C immunoprecipitation experiments, but not in identical anti-IgG immunoprecipitations are indicated (*arrows*, *right side*). n = 3 experiments in which distinct HASMC cell lysates were treated identically. *B* and *C*, representative high-resolution confocal images of HASMCs, transfected with either myc-Orai1, GFP-STIM1, HA-ADCY8, or FLAG-PDE1C plated on coated coverslips (*B*) or Fluoroblok membranes (*C*). Transfected HASMCs plated on coverslips were incubated with primary antibodies for individual expression tags (*i.e.*, myc, GFP, HA, FLAG) or with an anti-AKAP79 antibody. Staining of the expressed proteins, or of endogenous AKAP79, are shown in *green* while actin tetramethylrhodamine-isothiocyanate-conjugated phalloidin is shown in *red*. *C*, HASMCs, transfected with either myc-Orai1, GFP-STIM1, HA-ADCY8, or FLAG-PDE1C were plated on Fluoroblok membranes and allowed to accumulate LEPs on the underside of the membranes. LEP on the underside of Fluoroblok membranes were stained with anti-myc, anti-GFP, anti-HA, anti-FLAG, or anti-AKAP79; visualized by staining with 488-conjugated secondary for anti-myc, anti- GFP, anti-HA, anti-FLAG and anti-AKAP79 and tetramethylrhodamine-isothiocyanate-conjugated phalloidin to visualize F-actin and DAPI for nuclei; scale bars, 20 μm, n = 5 to 7 transfections were analyzed per condition. *Arrowheads* indicate staining of each protein and their accumulation within LEPs. ADCY, adenylyl cyclase; HASMC, human arterial smooth muscle cell; LEP, leading-edge protrusion; PDE, phosphodiesterase; PDE1C, phosphodiesterase 1C; STIM1, stromal interaction molecule 1.

Previous studies, largely using heterologous expression systems, indicated that Orai1, AKAP79, PKA, and ADCY8 could be co-immunoprecipitated from certain cell lysates (7, 9, 11, 14). Because our data indicated that PDE1C selectively regulated SOCE and SOCE-mediated ADCY8 activation in HASMCs, we investigated whether immunoprecipitation of PDE1C from HASMC lysates would allow recovery of a similar protein complex in these cells. Consistent with this, AKAP79, PKA-RIIβ, and Orai1 were each recovered, with PDE1C, from anti-PDE1C immune complexes (Fig. 6*A*). Potentially speaking to the selectivity of these interactions, anti-PDE1C immune isolates did not contain gravin, a distinct HASMC-expressed AKAP, STIM-1, or PKARIα (Fig. 6*A*). Although overexpressed HA-tagged ADCY8 could sometimes be recovered in anti-PDE1C isolates (not shown), very high levels of background associated with overexpression of this enzyme precluded confirming its presence in these complexes in these experiments.

Based on the finding that PDE1C, AKAP79, Orai1, PKA, and perhaps ADCY8 might interact in HASMCs, we next analyzed the distribution of these proteins in “static” cell cultures and in cultures in which polarized cells extend LEPs. Because the relatively low avidity of the primary antibodies available commercially prohibited their use in cell staining, these studies were carried out mostly with expressed epitope-tagged fusions of the relevant proteins. Staining of HASMC cultures indicated that myc-Orai1, HA-ADCY8, or AKAP79 each distributed broadly throughout these cells (Fig. 6*B*) and that GFP-STIM1 staining was largely, but not exclusively, perinuclear (Fig. 6*B*). Interestingly, although FLAG-PDE1C was detected throughout HASMCs, its staining was more abundant in cellular regions devoid of cell–cell contacts (Fig. 6*B*, *arrowheads*). When HASMC LEPs were immunostained, each of the proteins of interest were detected in these structures. Indeed, staining detected each AKAP79, myc-Orai1, and HA-ADCY8 in HASMC LEPs. Interestingly, staining for myc-Orai1 and HA-ADCY8 was relatively more abundant at the tips, than the bulk cytosol of LEPs (Fig. 6*C*, *arrowheads*). Consistent with the idea that ER(Ca^2+^) release occurs at leading edges of polarized and migrating cells, GFP-STIM1 staining was detected in LEPs (Fig. 6*C*). Most strikingly, while levels of FLAG-PDE1C staining was low in the bulk cytosol of HASMC LEPs, this staining was highly concentrated at the tips of these structures (Fig. 6*C*, *arrowheads*); a result suggesting that PDE1C concentrates at the tips of these structures. Although the dynamic nature of these structures (see Video S1) did not allow for live cell analysis of PDE1C accumulation in these structures, we hypothesize that the distribution of FLAG-PDE1C is consistent with the idea that PDE1C “marks” the expanding tips of these structures and, perhaps, leads the way during LEP formation in polarized HASMCs.

## Discussion

Ca^2+^ and cAMP signaling systems are each critically involved in regulating virtually all processes in mammalian cells, including their proliferation, motility, and intermediary metabolism (37, 38). Recent detailed studies, largely in heterologous expression systems, have established that SOCE, the primary mechanism of Ca^2+^ entry in nonexcitable cells, selectively modulates cellular cAMP synthesis in such cells (8, 11, 13, 15, 16). Mechanistically, these effects are largely coordinated through Ca^2+^/CaM-dependent activation of certain ADCYs variants (ADCY1 or ADCY8) or Ca^2+^-dependent, but CaM-independent, inhibition of others (ADCY5 or ADCY6) (7). In contrast, before this work, there had been no comprehensive study linking SOCE-directed regulation of ADCY-catalyzed cAMP synthesis and Ca^2+^activation of PDE-catalyzed cAMP hydrolysis.

Herein, we described results of studies aimed specifically at assessing whether the activity of the Ca^2+^/CaM-activated cAMP PDE expressed in HASMCs (*i.e.*, PDE1C) was regulated in the context of SOCE and the impact of this regulation on SOCE-ADCY crosstalk in HASMCs, a critical vascular cell type. These issues were studied by inhibiting, or silencing, PDE1C and monitoring how these interventions modified SOCE-dependent and SOCE-independent cAMP homeostasis in HASMCs. Our findings showed that increases in intracellular Ca^2+^, either resulting from ER(Ca^2+^) store depletion or SOCE, activated PDE1C and conclude that this PDE1C activation reflects, at least in part, Ca^2+^/CaM binding and activation. Because PDE1C activation survived cell lysis, we further predict that some PDE1C activation may reflect protein phosphorylation or some other stable modification. Of course, our future work will address the nature of this effect more formally. Importantly, however, we report here that PDE1C activation influenced the magnitude and rate of SOCE-mediated increases in intracellular Ca^2+^, and likely because of these effects, the ability of SOCE to regulate selectively the activities of each of the Ca^2+^-sensitive ADCYs expressed in these cells, namely ADCY6 and ADCY8. In relation to their impact on important cellular functions, our finding showed how PDE1C regulated formation of LEPs in polarized HASMCs, a critical early event required for cell migration.

Our findings build on earlier biochemical work in heterologous cell models (8, 11, 13, 15, 16) in which SOCE activation of ADCY8 was reported to occur largely within an AKAP79-based signaling complex that also was populated by Orai1, ADCY8, and PKA. In our work, we show that a similar macromolecular signaling complex containing AKAP79, Orai1, PKA, PDE1C, and perhaps ADCY8 likely also forms in HASMCs. In addition, we show that this superstructure integrates PDE1C and allows this PDE to directly and selectively regulate the activity of the anchored PKA that controls formation of LEPs in polarized HASMCs. Importantly, we also show that these events are coordinated downstream of SOCE-mediated activation of ADCY8. Interestingly, a recent report described a potential role for SOCE-associated changes in the activity of the soluble ADCY (ADCY10) in cells (39). Because our findings and those described in the work cited previously (8, 11, 13, 15, 16) indicate that SOCE-associated changes in cAMP are inhibited by transmembrane ADCY selective inhibitors, further work will be required to address the role of ADCY10, if any, in the processes described here.

Earlier work had unequivocally shown that crosstalk between SOCE and ADCY8-dependent cAMP signaling was coordinated locally within cellular membranes and likely within membranes enriched in lipid rafts (7, 11, 12), Here, we show that this system may localize selectively at leading edges of polarized HASMCs and could provide a platform through which cells can regulate hyperlocalized cellular events. Most importantly, our work is consistent with the novel idea that PDE1C and perhaps other proteins involved in this coordinated signaling are concentrated at the very tips of forming LEPs. Indeed, our cell staining data are entirely consistent with the idea that PDE1C localizes most preferentially at the tips of forming LEPs and suggest that it may guide their formation through hyperlocalized signaling at these tips. Owing to the dynamic nature of LEPs in live cells and their relatively small size, it was not possible to formally test this idea in nascent structures, but the absence of PDE1C from the bulk LEP volume is consistent with this idea.

Although our findings are most relevant to HASMCs, we predict that other cell types that express ADCY8 and PDE1C may avail themselves of the signaling dynamics reported here to efficiently regulate SOCE and coordinate localized effects of SOCE on ADCY8- and PKA-regulated functions. Indeed, certain nonexcitable cells, including macrophages and glioblastoma cells, also express Ca^2+^/CaM-activated PDEs (40, 41). Similarly, although not a focus of our work, recent evidence suggests that PDE1C inhibition could impact cAMP regulated cardiac myocyte contractility and that these may be less likely to promote arrhythmias than PDE3 inhibitors (42). Based on our work, we suggest that an analysis of how PDE1 inhibitors act in the heart and their role in regulating responses changes in Ca^2+^ signaling may be warranted. In this context, while few studies have addressed the need for SOCE in cardiac or skeletal myocytes, recent findings have suggested that SOCE may be involved in these cells and that this mode of Ca^2+^ entry may represent a novel therapeutic avenue worthy of further study (43). In addition, while our studies were focused exclusively on PDE1C, the dominant PDE1 family enzyme expressed in proliferative and migratory HASMCs, many cell types express other PDE1 family members (*i.e.*, PDE1A or PDE1B) including macrophages, cardiac, and neuronal cell types (44). Because PDE1A and PDE1B are largely cGMP-selective, it will be of significant interest to assess their roles in linking SOCE to changes in cGMP levels in these cells and to assess whether SOCE or ER(Ca^2+^) store depletion regulates their activities and cellular functions.

Conceptually, the potential significance of our work identifying Ca^2+^-dependent activation of PDE1C as a “trigger” that promotes optimal SOCE-mediated control of ADCY8 in HASMCs is likely best viewed in the context of recent work from Zhang *et al.* (16). Their work showed that SOCE-associated activation of ADCY8 facilitates PKA-mediated phosphorylation and inactivation of Orai1 during Ca^2+^-dependent inactivation. In this context, our work identifies PDE1C as a potential further component of this localized signaling system in cells. Indeed, our data are consistent with a role for PDE1C activation, after ER(Ca^2+^) store depletion, in recruiting the PKA-inhibited fraction of Orai1 into SOCE or perhaps in prolonging Orai1 opening during SOCE. Of course, PDE1C could subserve both these roles in cells. Thus, we suggest that our finding represents a critical further step in this process, one that represents a Ca^2+^-dependent system through which cells could reduce PKA phosphorylation of Orai1 and consequently promote recruitment of Orai1 into a “new” round of SOCE or extend its activity. Thus, with analogy to the process described previously (16), we suggest that PDE1C activation might represent a system of Ca^2+^-dependent reactivation of PKA-inhibited Orai1 (see Graphical Abstract).

## Experimental procedures

### Cells, cell cultures, and transient RNAi

Human internal thoracic artery smooth muscle cells were isolated from vessels used for coronary artery bypass graft surgery at the Kingston General Hospital (KGH) and processed as described in (45). On three occasions, HASMCs were also purchased from a commercial vendor (Cell Applications). In all, HASMCs derived from 37 different donors were used separately in the experiments described herein. Use of the human arterial tissues obtained from KGH was approved by the Queen’s University Health Sciences & Affiliated Teaching Hospitals Research Ethics Board for project SURG-334-15 “Endothelial cell function in human hearts” and abides by the Declaration of Helsinki principles. For RNAi, HASMCs were cultured in basal smooth muscle basal media (SMBM) containing Lipofectamine 3000 (Invitrogen) and the desired siRNA at a 1:1 ratio. Media was changed 5 h posttransfection with smooth muscle growth media-2, and experiments were conducted 48 h posttransfection. The individual siRNAs used in these studies are listed in Table S1.

### cAMP PDE activity assay

PDE-catalyzed cAMP hydrolysis (*i.e.*, PDE activity) was determined as previously described (22, 46). The fraction of cAMP that was hydrolyzed by *PDE1*-, *PDE3*-, or *PDE4*-family–derived enzymes was determined using PDE-family selective inhibitors. To inhibit PDE1C activity, reactions were supplemented with either “Compound 33” (C33, 1 μM), a gift from Dr James Guy Breitenbucher (Dart Neuroscience) or PF-04827726 (1 μM), Sigma. To inhibit PDE3 or PDE4 activities, cilostamide (5 μM), Cedarlane, or Ro, 20 to 1724 (10 μM), Calbiochem, were used, respectively.

### Immunoprecipitations and immunoblotting

To immunoprecipitate endogenous PDE1C, HASMCs were grown to confluent monolayers, and lysates were collected using triton-based lysis buffer: 1.0% Triton X-100, 100 mM sodium pyrophosphate, 10 mM sodium β-glycerophosphate, 5 mM benzamidine, 10 mM sodium orthovanadate, 50 mM Tris-HCl, 100 mM sodium chloride, 1 mM EDTA, 5 mM magnesium chloride, 0.5 mM calcium chloride, and 10 mM PMSF and the following protease inhibitors: 1 μg/ml pepstantin A, 1 μg/ml E-64, 5 μg/ml bestantin, 1 μg/ml aprotinin, 2 μg/ml leupeptin. Lysates were homogenized (20G needle), centrifuged at 10,000 RPM, and a fraction of the supernatant was collected for analysis of total lysate (input). To reduce nonspecific binding, lysates were precleared with protein A/G plus beads (40 μl bed volume: Santa Cruz) for 3 h with anti-IgG goat (1 μg/ml: Santa Cruz). Following centrifugation (5000 RPM), lysates were collected and immunoprecipitated with 1 μg/ml of anti- PDE1C (Santa Cruz) with protein A/G plus beads (40 μl bed volume) for 16 h at 4 °C. The beads were washed 3X with Triton lysis buffer, and protein was eluted at 37 °C for 30 min followed by immunoblotting. Antibodies and working concentrations for immunoblotting are indicated in the Table S2. Anti-PDE1C (Fabgenix) was used for immunoblotting to determine the knockdown efficiency of PDE1C following 48 h siRNA treatment, and anti-PDE1C (Santa Cruz) was used for immunoblotting of PDE1C following immunoprecipitation of PDE1C.

### Assay of leading-edge protrusion dynamics

HASMCs resuspended in SMBM were plated on the upper surface of gelatin-coated [ddH2O supplemented with 0.25% gelatin (Biorad)], 24 mm^2^-diameter BD Falcon FluoroBlok cell culture inserts forming a monolayer (3 μm) to investigate leading edge protrusion as previously conducted (47, 48). Chemotaxis was initiated by adding 0.5% FBS to the underside of the inserts to allow cells to form leading edge protrusions or migrate for 4 h. Pharmacological activators or inhibitors were added to the top of the insert before the addition of FBS to the underside of the inserts. The following drugs were used: forskolin (Sigma), PKI [14 22] myristylated PTD (ThermoFisher), StHt31P and StHT31 (Promega), atrial natriuretic peptide (ANP) human (Sigma), and the following PDE inhibitors, C33, PF-04827726, cilostamide, and Ro 20 to 1724. To visualize the extent of LEP, inserts were fixed with paraformaldehyde [4% (vol/vol)], rinsed with Hank's balanced salt solution (HBSS), and incubated for 1 h with phalloidin tetramethylrhodamine B isothiocyanate (1:1000; Sigma) and 4′,6-diamidino-2-phenylindole (DAPI) (1:1000; Thermofisher) (0.3% bovine serum albumin (BSA) diluted in HBSS). Inserts were mounted on glass slides, and the density of LEPs was quantified by measuring the total fluorescence of tetramethylrhodamine-isothiocyanate-phalloidin on the bottom of the insert by imaging 4 to 5 quadrants in four transwells per condition, per experiment. Images were obtained with a Zeiss Axiovert S100 microscope and imaged with Slidebook software. Visualization of real-time LEPs was conducted by transducing HASMCs with the LifeAct-TagGFP2 adenovirus with an multiplicity of infection of 1000. Following 72 h infection, HASMCs were plated on 24 mm^2^-diameter (3 μm) BD Falcon cell culture inserts and imaged as indicated above.

### Visualization of proteins in whole cell bodies and the leading edge

Protein localization in leading edge protrusions or cell bodies was visualized by transiently transfecting a monolayer of HASMCs with 2 μg of DNA plasmids (Table S2) (myc- Orai1, GFP-STIM1, FLAG-PDE1C, GFP-PDE3B, and GFP-PDE4D7) using TransfeX (ATTC) as recommended by the manufacturer’s protocol and plating cells on 3 μm inserts 48 h posttransfection. HA-ADCY8 adenovirus (Abgood) was infected in HASMCs with an multiplicity of infection of 0.1 using Ibidi Boost (Ibidi) according to the manufacturer’s protocol and plating cells on 3 μm 24 mm^2^-diameter BD Falcon cell culture inserts 72 h postinfection. Following plating, HASMCs on inserts in SMBM containing media, 0.5% FBS diluted in SMBM was added to the bottom of the transwell, and cells were allowed to extend leading edge protrusions for 4 h. Specific proteins were visualized by fixing the inserts with paraformaldehyde [4% (vol/vol)], rinsed with HBSS, and incubated for 1 h with the following primary antibodies: anti-c-myc mouse monoclonal (Sigma 1:1000), anti-GFP monoclonal rabbit (Santa Cruz 1:1000), anti-Flag M2 monoclonal mouse (Sigma 1:1000), for 1 h at room temperature or anti-AKAP79 rabbit polyclonal (Upstate Cell Signaling Solutions 1:100) at 4 °C for 16 h. The inserts were then washed with HBSS and incubated with fluorescently labeled Alexa-conjugated (488 nm) secondary antibodies and with phalloidin-tetramethylrhodamine B isothiocyanate (1:1000) and DAPI (1:1000) (0.3% BSA diluted in HBSS). The inserts were mounted on glass slide, and protein localization at the leading edge was visualized using a Leica TCS SP8 confocal laser scanning microscope.

### Fura-2 Ca^2+^ imaging

Measurement of [Ca^2+^]_i_ in HASMCs was performed using the ratiometric Ca^2+^ indicator Fura-2 AM, as described previously (49). Fura-2 AM (kept in the dark at −20 °C; Invitrogen) was dissolved in DMSO to a concentration of 1 mg/ml. Pluronic F-127 (Invitrogen; 0.5 μl/μl of DMSO) was added, and the resulting solution was briefly vortexed. HASMCs were loaded with 5 μM Fura-2 AM in Krebs solution containing 125 mM NaCl, 5 mM KCl, 1 mM Na_2_HPO4, 1 mM MgCl_2_, 5.6 mM Glucose, 20 mM Hepes, and 2 mM CaCl_2_ pH, 7.40, for 30 min at room temperature. For Ca^2+^ free solutions, the CaCl_2_ was omitted, and EGTA (25 μM) was added to the buffer. The cells were then washed with Krebs and kept at room temperature for an additional 30 min before imaging. Fluorescence emitted from Fura-2 AM was captured with an InCyt dual-wavelength imaging system (Intracellular Imaging) and a PixelFly CCD camera (1360 × 1024 resolution) mounted on a Nikon Eclipse TS100 (Nikon). HASMCs were perfused at a flowrate of 3 ml/min and were allowed to equilibrate for >5 min before data collection. Data were collected at 0.167 Hz. Cell viability was determined by a brief (15 s) application of 5 μM ionomycin (Sigma) at the end of the experiment. The change in the ratio induced during store depletion by 10 μM cyclopiazonic acid (CPA; Sigma) in Ca^2+^ free Krebs buffer, and the change in the ratio induced by SOCE was determined as the difference between the peak during Ca^2+^ free Krebs + CPA and the trough before bath application of Ca^2+^ free Krebs + CPA and the difference between the peak during Krebs + CPA and the trough before bath application of Krebs + CPA, respectively. The rate in change during store depletion (Ca^2+^ free + CPA) and SOCE (Krebs + CPA) was determined as the difference in the ratio value between the peak drug response and the trough before drug response/the time difference between the point at which the drug has reached the peak response and the trough time point before drug response.

### Fluorescence resonance energy transfer imaging of cAMP and PKA

FRET-based measurements of cAMP or PKA in HASMCs were carried out as follows: HASMCs were transiently transfected with the mTurq2ΔEPAC^cp173^Ven_Ven sensor (a gift from Jalink Kees: Lab ID Epac-SH134) for the detection of cAMP activity or with pcDNA3-AKAR4 sensor (a gift from Jin Zhang; Addgene plasmid #61619) for the detection of PKA activity using TransfeX (ATCC) in accordance with manufacturer’s instructions. Following transfection, cells were plated on glass coverslips coated with gelatin (0.25%) and imaged at room temperature 24 h posttransfection. H134-based measurements of changes in cAMP in the primary HASMCs used in our experiments were variable. To reduce the impact of this variability seen in responses of control or treated cells on our data, all experiments in which the effects of drugs or of silencing of genes of interest were conducted were carried out on the same day, with cells of the same passage number. In addition, when representative traces are shown, the individual traces being compared were obtained in experiments carried out at the same time with cells of the same passage number. Real-time FRET was performed using the Leica DMi8 inverted microscope equipped with a HC PL FLUOTAR 40×/1.30 oil immersion objective, a Leica EL6000 light source and a C11440 ORCA- Flash 4.0 digital camera (Hamamatsu). The following solutions were incubated in HASMCs to perform the SOCE protocol; Krebs, Ca^2+^ free Krebs for 5 min to capture a baseline, addition of CPA (10 μM) for 5 min, followed by Ca^2+^ containing Krebs with CPA (10 μM) for 10 min, followed by saturation of the cAMP or PKA probe with 10 μM forskolin (Sigma), and 100 μM 3- Isobutyl-1-methylxanthine (Calbiochem). Three filter sets (CHROMA) were used to acquire images: for CFP, excitation filter 430/424 nm, emission 470 nm; for FRET (CFP/YFP) cube, excitation 430/24 nm, DC 440 nm; 520 nm, emission 540 nm, for YFP, excitation 500/520 nm, emission 535 nm. Images were acquired every 5 s with an exposure of 150 to 449 ms and processed using LAS X Version 2.0.0.14332 software (Leica). FRET-based measurements were quantified by defining a region of interest (ROI) per whole cell. FRET was measured in the selected ROI of each image acquired by capturing fluorescence in three channels: CFP for direct donor excitation and emission, YFP-FRET for donor-sensitized acceptor emission, and YFP for direct acceptor excitation and emission. Calculation of the FRET efficiency was determined by performing a background correction in each fluorescence channel captured by subtracting the background fluorescence intensity in a ROI that contained no cells from the emission intensity from the cells expressing the biosensor. FRET emission ratios (YFP-FRET/CFP) were calculated for each time point and normalized over the time course by dividing the emission ratio at each time point by the value preceding drug application. Data are presented as single representative tracings from experiments using individual cells. Averaged data are shown as mean changes in FRET, where these were obtained, in all cases, by determining the maximal peak response following drug application from the preceding baseline. Initial rates of SOCE activation-associated cAMP increase were derived from calculations of the slope between, (a) the apex of the initial SOCE-associated increase in FRET (“area B” in Fig. 1*B*) and (b) a point 100 s after the apex.

### RNA isolation, reverse transcription, and qPCR

HASMC RNA was isolated using the Qiagen RNeasy (Qiagen) mini kit as per manufacturer’s instructions. The purity and the quantity of RNA isolated was determined using a Nanodrop 1000 (Thermo Scientific). cDNA was synthesized from this RNA using a Qiagen Omniscript RT according to the manufacturer’s instructions. qPCR reactions were performed using PowerUP SYBR Green Master Mix (Thermo Fisher Scientific) with 2 ng cDNA template and the primers detailed in Table S3. Thermocycler conditions were the following using the QuantStudio 5 Real-Time PCR System: PCR Stage: Step 1, 95 °C and 15 min; Step 2, 60 °C and 1 min repeated 40X and Melt Curve Stage: Step 1, 95 °C and 15 min; Step 2, 60 °C and 1 min; Step 3, dissociation 95 °C and 1 s.

### Puncta visualization and quantification of STIM1 to ORAI translocation

HASMCs were transfected with myc-ORAI1 and GFP-STIM1 using 2 μg of DNA using the HASMC nucleofector kit (Lonza) according to the manufacturer’s instructions and the Nucleofector 2b Device (Lonza). Following 24 h post-DNA transfection, cells were plated on gelatin-coated coverslips (ddH_2_O supplemented with 0.25% gelatin), and following 48 h post-DNA transfection, cells were subjected to Krebs buffer for 5 min, followed by store depletion (Ca^2+^ free + CPA 10 μM) for 5 min. After treatment with Krebs, or store depletion, cells were fixed using paraformaldehyde [4% (vol/vol)], rinsed with HBSS and incubated for 1 h at room temperature with the following primary antibodies: anti-c-myc mouse monoclonal (Sigma 1:1000) and anti-GFP monoclonal rabbit (Santa Cruz 1:1000). The coverslips were then washed with HBSS and incubated with fluorescently labeled Alexa-conjugated (488 nm) secondary antibodies and with phalloidin-tetramethylrhodamine B isothiocyanate (1:1000) and DAPI (1:1000) (0.3% BSA diluted in HBSS). Imaging was performed using the Leica TCS SP8 confocal microscope (Leica). White light laser (WLL) system was adjusted with settings for the laser line 499 nm (excitation of Alexa-fluor 488; GFP-STIM1) and 572 nm (excitation for Alexa-fluor 568; myc- Orai1) and UV laser (405 nm) to identify DAPI nucleolus. Individual cells were imaged through a z-stack (Z-dimension of 2–3 μm; pixel size of 0.29 μm) acquisition mode using the HC PL APO CS2 63×/1.40 oil objective. The analysis was performed with the LAS-X Software (Leica Microsystems). To measure the relative mobilization distance of GFP-STIM1 to myc- Orai1 (cell surface), three straight ROIs were drawn per cell initiating it at the central point of each nucleolus and extending to the last bright point representing the cell surface. The intensity of each marker (DAPI, GFP-STIM1, and myc-Orai1) was measured through the ROI (see Fig. S3). Any intensity peak lower than 5000 (AU) was considered baseline background. Length measurements of the last peak (ρ) of DAP (ρDAPI), GFP-STIM1 (ρSTIM1), and myc-Orai1(ρOrai1) were taken, and the following formula was applied to obtain the relative STIM1 distance to Orai1. The relative STIM1 distance (RdSTIM1) equals 1 was suggestive of STIM1 trafficking to the cell surface.

### Statistical analysis

All data were analyzed statistically using GraphPad Prism Software. Throughout the manuscript, data are presented as means ± SEM. Statistical differences between two groups were obtained from unpaired, two-tailed Student’s *t* tests. When multiple groups were compared, statistical significance was analyzed using either a 1- or a 2- way analysis of variance, as appropriate, followed by an appropriate post hoc test as indicated in individual figure captions. In this manuscript, *p* ≤ 0.05 was considered significant.

## Data availability

All the data are contained within the manuscript and supplemental information.

## Supporting information

This article contains supporting information (27, 28, 29, 30, 31, 32, 33).

## Conflict of interest

None declared.
