# Supplementary material for: Phosphodiesterase 1C integrates store-operated calcium entry and cAMP signaling in leading-edge protrusions of migrating human arterial myocytes
Source: J Biol Chem. 2021 Mar 28;296:100606. doi: 10.1016/j.jbc.2021.100606 (PMC8095186; doi:10.1016/j.jbc.2021.100606)
Supplement: Supplemental Figures S1–S9 and Tables S1–S4 [file mmc1.pdf]

Supplementary Material

Phosphodiesterase 1C integrates store-operated calcium entry and cAMP signaling in leading-edge protrusions of migrating human arterial myocytes

Paulina Brzezinska[1], Nicholas J. Simpson[1], Fabien Hubert[1], Ariana N. Jacobs[1], M. Bibiana Umana[1], Jodi L. MacKeil[1], Jonah Burke-Kleinman[1], Darrin M. Payne[2], Alastair V. Ferguson[1] and Donald H. Maurice[1]

Department of Biomedical and Molecular Sciences[1] and Department of Surgery[2] Queen's University, Kingston, Ontario, Canada, K7L 3N6

Supplementary Reagents

Supplementary Table 1. Target genes and siRNA sequences

Target	siRNA ID	Sense	Antisense	Source
Control	Silencer select negative control No.1 siRNA	N/A commercial product		Thermofisher
PDE1C #1	PDE1CHSS182019	5'-CACCAGCUGUU AUUGAGGCAUUAAA-3'	5'-UUUAAUGCCUCA AUAACAGCUGGUG-3'	Invitrogen
PDE1C #2	PDE1CHSS107703	5'-UAUAGCAAAGAU CUCCAGCUCCGUC-3	5'-CACCAGCUGUUA UUGAGGCAUUAAA-3'	Invitrogen
ADCY6	J-006636-06, J-006636-07, J-006636-08, J-006636-09	5'-GUGAAUGUCUCUAGUCGUA-3', 5'-CCACAUCACUCGGGCAACA-3', 5'-GUCCUUGGCUUGCGGAAU-3', 5'-GUGGUUCUCUGUUCCCUAA-3',		Dharmacon (ON-TARGETplus)
ADCY8	S1036	5'-GCUGUAUUCU CAAUCCUAUtt-3'	5'-AUAGGAUUGA GAAUACAGCtc-3'	Ambion
PKA	PRKACAVHS50343	5'-GGAAGCUCCCU UCAUACCAAAGUUU-3'	5'-AAACUUUGGUAU GAAGGGAGCUUCC-3'	Invitrogen

Supplementary Table 2. Reagents and resources

Antibodies	Source	Identifier	Working Concentration
Rabbit polyclonal anti-PDE1C	Fabgenix	PD1C-301AP	1:1000
Goat polyclonal anti-PDE1C	Santa Cruz	T-17:sc-54939	1:500
Mouse monoclonal anti-PKAc	BD Biosciences	610980	1μg/ml
Rabbit polyclonal anti-AKAP79	Upstate Cell Signaling Solutions	07-235	1:5000
Rabbit polyclonal anti-ADCY8	Proteintech	55065-1-AP	1:500
Mouse monoclonal anti-RIIβ	BD Transduction Laboratories	610625	1:1000
Rabbit monoclonal RI-α	Cell Signaling	D54D9	1:1000

Goat polyclonal anti-Gravin	Abcam	Ab9698	1:1000
Rabbit polyclonal anti-STIM1	Cedarlane	A303414am	1:1000
Goat polyclonal anti-Orai1	Novus biologicals	Nbp1-06992	1:300
Rabbit anti-GFP	Thermofisher	A11122	1:1000
Mouse anti-c-myc	Sigma-Aldrich	m-4439	1:1000
Mouse monoclonal anti-HA	Santa Cruz	(F-7) sc-7392	1:1000
Mouse anti-FLAG M2	Sigma-Aldrich	F3165	1:1000
Mouse monoclonal anti Beta Actin	Sigma-Aldrich	A5441	1:10000
anti-Beta Tubulin	Sigma-Aldrich	T5293	1:1000

**Chemical and peptides**	**Source**	**Identifier**
Collagenase from Clostridium histolyticum lyophilized powder	Sigma-Aldrich	C9722
Lipofectamine 3000	Invitrogen	L3000015
Crotalus atrox snake venom	Creative Enzymes	NATE-0072
[^{3}H]-cyclic AMP	Perk in Elmer	2016103
[[$^{8\text{-}3}$ H] Hypoxanthine	Americcan Radiolabeled Chemicals Inc	ART 0266
Cilostamide	Calbiochem	231085
Ro-20-1724	Calbiochem	557502
C33	Generous gift from James Guy Breitenbucher (Dart Neuroscience)	N/A
PF-04827726	Sigma	Pz0379
Forskolin	Sigma	F3917
8-Methyoxymethyl-3-isobutyl-1-methylxanthine (IBMX)	Calbiochem	454202
Ca^{2+}/Calmodulin Solution 10X	Abcam	Ab189137
PKI (1422) myristylated PTD	Thermofisher	77409
St-Ht31	Promega	V821A
St-Ht31 P	Promega	V822A
Cyclopiazonic Acid (CPA)	Sigma	C1530
Fura-2AM	Invitrogen	F1201
Pluronic F-127	Invitrogen	P3000MP
Ionomycin	Sigma-Aldrich	10634
EGTA	ICN Biomedicals	195174
Gelatin	BioRad	1706537
atrial natriuretic peptide (ANP) human	Sigma-Aldrich	A1663
Sildenafil	Extracted from commercially available Viagra® tablets as described in *Proc Natl Acad Sci U S A.* 105(36), 13650-5 (2008)	N/A
SQ 22536	Sigma	S153
Phalloidin	Sigma	P1951
DAPI	Thermofisher	D3571
PowerUP™ SYBR™ Green Master Mix	Thermofisher	A25742
normal goat IgG	Santa Cruz	Sc-2028

**Recombinant DNA or virus**	**Source**	**Identifier**
myc-ORAI1	Anjana Rao, Addgene	Plasmid # 21638
CFP-STIM1	Tobias Meyer, Addgene	Plasmid #18858
FLAG-PDE1C	Generous gift from Chen Yan	N/A
GFP-PDE3B	Generous gift from Vincent Manganiello	N/A
GFP-PDE4D7	Generous gift from George Baillie	N/A
mTurq2ΔEPACcp173Ven_Ven	Generous gift from Jalink Kees	N/A
pcDNA3-AKAR4	Jin Zhang, Addgene	Plasmid #61619
HA-ADCY8 adenovirus	Abgood	067660A
LifeAct-TagGFP2 adenovirus	Ibidi	60121

**Commercial Kits**	**Source**	**Identifier**
DEAE–Sephadex® A-25 beads	Sigma	A25120
Human Aortic Smooth Muscle Cell NucleofectorTM Kit	Lonza	VAPC-1001
Protein A/G Plus beads	Santa Cruz	Sc-2003
Qiagen RNeasy Kit	Qiagen	74104
Qiagen Omniscript RT	Qiagen	205111

**Transfection and Transduction Reagents**	Source	Identifier
Lipofectamine 3000	Invitrogen	L3000015

TransfeX™ Transfection Reagent	ATCC	ACS-4005
ibiBoost Adenovirus Transduction Enhancer	Ibidi	50301

Supplementary Table 3. **Primers for real-time qPCR**

Target	Sense	Antisense
PDE1C	5'-CAGCAAAAGCATGGGACCTC-3'	5'-TGAAGGTGGGTTCCACGATG-3'
PKAcα	5'-ATGTTCTCACACCTACGGCG-3'	5'-CCAGCGAGTGCAGATACTCA-3'
ADCY6	5'-GAGATCATCGCTGACTTTGATG-3'	5'-TCGTAGGTGCTGGCGTTC-3'
ADCY8	5'-TCAAGCCATTCTCACTGATGTT-3'	5'-TGAACACTTCATCCCTCATTTG-3'
TBP	5'-TATAATCCCAAGCGGTTTGC-3'	5'-GCTGGAAAACCCAACTTCTG-3'
PGK	5'-CTGTGGGGGTATTTGAATGG-3'	5'-CTTCCAGGAGCTCCAAACTG-3'

Supplementary Methods

Cell Culture and siRNA Transient Transfections

Human internal thoracic artery smooth muscle cells (HASMCs) were isolated from discarded unused portions in coronary artery bypass graft surgeries as described by Moss and colleagues [27] from donor patients of Kingston General Hospital as well as purchased from Cell applications. For tissues obtained from KGH, their use in this research study (SURG-334-15; "Endothelial cell function in human hearts") was approved by the Queen's University Health Sciences & Affiliated Teaching Hospitals Research Ethics Board (HSREB). HASMCs were cultured in smooth muscle basal medium (SMBM) and smooth muscle growth medium bullet kit (SMGM-2) (Lonza), supplemented with 10% FBS, cultured at 37°C in 5% CO_2 and used between passages 4-9. The received tissue was rinsed and maintained in HBSS. The artery was incubated in 2mg/mL of type 1 collagenase in HBSS for 15 min at 37°C and the adventia was then removed. The artery was cut longitudinally, and the endothelium were manually scraped off. The artery was further cut into 1-2 mm^3 pieces and digested in new 2 mg/mL type 1 collagenase solution in HBSS for 1-2 h at 37°C. The tissue was then centrifuged at 1500 RPM for 10 min, and pelleted SMCs were collected and seeded in a $25cm^2$ flask containing SMGM-2 supplemented with 10% FBS. For siRNA transfection, HASMCs were cultured in basal SMBM containing Lipofectamine 3000 (Invitrogen) and siRNA in a 1:1 ratio and media was changed 5 h post transfection with SMGM-2. Experiments were conducted 48 h post transfection. The following siRNAs used are indicated in **Supplementary Table 1**.

cAMP PDE Activity Assay

cAMP PDE enzymatic activity was determined by a two-step radioenzymatic assay as previously described [28, 29]. Individual reactions were performed in a total volume of 250 µl containing the following; 1 µM [^{3}H]-cyclic AMP (100 000 dpm) in a solution containing; 20 mM Tris, 20 mM Imidazole, 3 mM $MgCl_2$, 0.2 mg/mL BSA, 4 µg/mL calmodulin, 0.04 mM $CaCl_2$ pH 7.5, with the following PDE inhibitors: PDE3 inhibitor (Cilostamide 5 µM; Cedarlane), PDE4 inhibitor (Ro 20-

1724 10 µM; Calbiochem), PDE1 inhibitor (Compound C33 (C33) 1 µM; generous gift from Dr. James Guy Breitenbucher (Dart Neuroscience), and PDE1 inhibitor (PF-04827726 1 µM; Sigma). The reaction was initiated by incubation of samples at 30°C for 30 min and terminated by boiling the reaction for 1 min. 5'AMP product was converted to adenosine by the addition of snake venom. The samples were run through DEAE–Sephadex® A-25 beads (Sigma) packed in chromatography columns, using a low salt buffer to elute samples off the column. The eluted samples were diluted with scintillation cocktail fluid (50%; Fisher) and measured using the LS 6500 Multi-Purpose Scintillation Counter.

cAMP activity assay

Confluent monolayers of HASMCs were incubated overnight with [^{3}H]-hypoxanthine to label intracellular cyclic nucleotide metabolic pools as described previously [30]. After removal of labeling media, cells were resuspended in SMBM and plated on 0.25% gelatin coated wells and incubated (37°C, 5% CO_2) for 2 h. HASMCs were treated with forskolin or received no treatment for 1 min. Reactions were terminated by TCA. cAMP was isolated and purified via column chromatography and [^{3}H]-cAMP was determined using the LS 6500 Multi-Purpose Scintillation Counter.

Chemotactic leading edge protrusion assays

HASMCs resuspended in SMBM basal media were plated on the upper surface of gelatin-coated ((ddH2O supplemented with 0.25% gelatin (Biorad)), 24 mm^2-diameter BD Falcon Fluoroblok™ cell culture inserts forming a monolayer (3 µm) to investigate leading edge protrusion as previously conducted [31, 32]. Chemotaxis was initiated by adding 0.5% FBS to the underside of the inserts to allow cells to form leading edge protrusions or migrate for 4 h. Pharmacological activators or inhibitors were added to the top of the insert prior to the addition of FBS to the underside of the inserts. The following drugs were used: forskolin (Sigma), PKI (1422) myristylated PTD (Thermofisher), st-Ht31P and st-HT31 (Promega), atrial natriuretic peptide (ANP) human (Sigma), and the following PDE inhibitors, C33, PF-04827726, cilostamide and Ro 20-1724. To visualize the extent of leading edge protrusion, inserts were fixed with paraformaldehyde (4% (vol/vol)), rinsed with HBSS and incubated for 1 h with phalloidin-tetramethylrhodamine B isothiocyanate (1:1000; Sigma) and DAPI (1:1000; Thermofisher) (0.3% BSA diluted in HBSS). Inserts were mounted on glass slides and the density of leading edge protrusions was quantified by measuring the total fluorescence of phalloidin-TRITC on the bottom of the insert by imaging 4-5 quadrants in 1-3 transwells per condition, per experiment. Images were obtained with a Zeiss Axiovert S100 microscope and imaged with Slidebook software. Visualization of real-time leading edge protrusions was conducted by transducing HASMCs with the LifeAct-TagGFP2 adenovirus with an MOI of 1000. Following 72 h infection, HASMCs were plated on 24 mm^2-diameter (3 µm) BD Falcon cell culture inserts and imaged as indicated above.

Visualization of proteins in whole cell bodies versus the leading edge

Protein localization in leading edge protrusions or cell bodies was visualized by transiently transfecting a monolayer of HASMCs with 2 µg of DNA plasmids (**Supplementary Table 2**) (myc-Orai1, GFP-STIM1, FLAG-PDE1C, GFP-PDE3B, GFP-PDE4D7) using TransfeX™

(ATTC) as recommended by the manufacturer's protocol and plating cells on 3 µm inserts 48 h post transfection. HA-ADCY8 adenovirus (Abgood) was infected in HASMCs with an MOI of 0.1 using Ibidi Boost (Ibidi) according to the manufacturer's protocol and plating cells on 3 µm 24 mm^2-diameter BD Falcon cell culture inserts 72 h post infection. Following plating HASMCs on inserts in SMBM containing media, 0.5% FBS diluted in SMBM was added to the bottom of the transwell, and cells were allowed to extend leading edge protrusions for 4 h. Specific proteins were visualized by fixing the inserts with paraformaldehyde (4% (vol/vol)), rinsed with HBSS and incubated for 1 h with the following primary antibodies: anti-c-myc mouse monoclonal (Sigma 1:1000), anti-GFP monoclonal rabbit (Santa Cruz 1:1000), anti-Flag M2 monoclonal mouse (Sigma 1:1000), for 1 h at room temperature or anti-AKAP79 rabbit polyclonal (Upstate Cell Signaling Solutions 1:100) at 4°C for 16 h. The inserts were then washed with HBSS and incubated with fluorescently-labelled Alexa-conjugated (488 nm) secondary antibodies and with phalloidin-tetramethylrhodamine B isothiocyanate (1:1000) and DAPI (1:1000) (0.3% BSA diluted in HBSS). The inserts were mounted on glass slides and protein localization at the leading edge was visualized using a Leica TCS SP8 confocal laser scanning microscope.

Fura-2 Ca^{2+} imaging

Measurement of $[Ca^{2+}]_i$ in HASMCs was performed using the ratiometric Ca^{2+} indicator Fura-2 AM, as described previously [33]. Fura-2 AM (kept in the dark at -20°C; Invitrogen) was dissolved in DMSO to a concentration of 1 mg/mL. Pluronic F-127 (Invitrogen; 0.5 µL/µL of DMSO) was added and the resulting solution was briefly vortexed. HASMCs were loaded with 5 µM Fura-2 AM in Krebs solution containing (in mM): 125 NaCl, 5 KCl, 1 Na_2HPO4, 1 $MgCl_2$, 5.6 Glucose, 20 Hepes and 2 $CaCl_2$ pH, 7.40, for 30 min at room temperature. For Ca^{2+} free solutions, the $CaCl_2$ was omitted and EGTA (25 µM) was added to the buffer. The cells were then washed with Krebs and kept at room temperature for an additional 30 min prior to imaging. Fluorescence emitted from Fura-2 AM was captured with an InCyt dual-wavelength imaging system (Intracellular Imaging) and a PixelFly CCD camera (1360x1024 resolution) mounted on a Nikon Eclipse TS100 (Nikon). HASMCs were perfused at a flowrate of 3 ml/min and were allowed to equilibrate for >5 minutes prior to data collection. Data was collected at 0.167 Hz. Cell viability was determined by a brief (15s) application of 5 µM ionomycin (Sigma) at the end of the experiment. The change in the ratio induced during store depletion by 10 µM cyclopiazonic acid (CPA; Sigma) in Ca^{2+} free Krebs buffer and the change in the ratio induced by SOCE was determined as the difference between the peak during Ca^{2+} free Krebs + CPA and the trough prior to bath application of Ca^{2+} free Krebs + CPA and the difference between the peak during Krebs + CPA and the trough prior to bath application of Krebs + CPA, respectively. The rate in change during store depletion (Ca^{2+} free + CPA) and SOCE (Krebs + CPA) was determined as the difference in the ratio value between the peak drug response and the trough before drug response/ the time difference between the point at which the drug has reached the peak response and the trough time point prior to drug response.

Fluorescence Resonance Energy Transfer (FRET) imaging cAMP and PKA

FRET-based measurements of cAMP or PKA in HASMCs were carried out as follows: HASMCs were transiently transfected with the mTurq2ΔEPAC^{cp173}Ven_Ven sensor (a gift from Jalink Kees: Lab ID Epac-SH134) for the detection of cAMP activity and HASMCs were transiently transfected with the pcDNA3-AKAR4 sensor (a gift from Jin Zhang; Addgene plasmid #61619) for the

detection of PKA activity using TransfeX (ATCC) in accordance with manufacturer's instructions. Following transfection, cells were plated on glass coverslips coated with gelatin (0.25%) and imaged at room temperature 24 h post transfection. Real-time FRET was performed using the Leica DMi8 inverted microscope equipped with a HC PL FLUOTAR 40×/1.30 oil immersion objective, a Leica EL6000 light source and a C11440 ORCA-Flash 4.0 digital camera (Hamamatsu). The following solutions were incubated in HASMCs to perform the SOCE protocol; Krebs, Ca^{2+} free Krebs for 5 min to capture a baseline, addition of CPA (10 µM) for 5 min, followed by Ca^{2+} containing Krebs with CPA (10 µM) for 10min, followed by saturation of the cAMP or PKA probe with 10 µM forskolin (Sigma) and 100 µM 3-Isobutyl-1-methylxanthine (Calbiochem). Three filter sets (CHROMA) were used to acquire images: for CFP, excitation filter 430/424 nm, emission 470 nm; for FRET (CFP/YFP) cube, excitation 430/24 nm, DC 440 nm; 520 nm, emission 540 nm, for YFP, excitation 500/520 nm, emission 535 nm. Images were acquired every 5 s with an exposure of 150-449 ms and processed using LAS X Version 2.0.0.14332 software (Leica). FRET-based measurements were quantified by defining a region of interest (ROI) per whole cell. FRET was measured in the selected ROI of each image acquired by capturing fluorescence in three channels; CFP for direct donor excitation and emission, YFP-FRET for donor-sensitized acceptor emission and YFP for direct acceptor excitation and emission. Calculation of the FRET efficiency was determined by performing a background correction in each fluorescence channel captured by subtracting the background fluorescence intensity in a ROI that contained no cells from the emission intensity from the cells expressing the biosensor. FRET emission ratios (YFP-FRET/CFP) were calculated for each time point and normalized over the time course by dividing the emission ratio at each time point by the value preceding drug application. Data is presented as single representative tracings from individual cells. Mean changes in FRET were obtained by determining the maximal peak response following drug application from the preceding baseline and mean changes in the change in rate were determined by quantifying the slope from time of drug application to the time at which the drug response plateaus.

Immunoprecipitations and western blotting

To immunoprecipitate endogenous PDE1C, HASMCs were grown to confluent monolayers and lysates were collected using triton based lysis buffer in mM: 1.0% Triton X-100, 100 sodium pyrophosphate, 10 sodium β- glycerophosphate, 5 benzamidine, 10 sodium orthovanadate, 50 Tris-HCl, 100 sodium chloride, 1 EDTA, 5 magnesium chloride, 0.5 calcium chloride, 10 PMSF, and the following protease inhibitors in µg/ml: 1 pepstantin A, 1 E-64, 5 bestantin, 1 aprotinin, 2 leupeptin. Lysates were homogenized (20G needle), centrifuged at 10 000 RPM and a fraction of the supernatant was collected for analysis of total lysate (input). To reduce nonspecific binding, lysates were precleared with Protein A/G Plus beads (40µl bed volume: Santa Cruz) for 3 h with anti-IgG goat (1µg/ml: Santa Cruz). Following centrifugation (5000 RPM), lysates were collected and immunoprecipitated with 1µg/ml of anti-PDE1C (Santa Cruz) with Protein A/G Plus beads (40µl bed volume) for 16 h at 4°C. The beads were washed 3X with triton lysis buffer and protein was eluted at 37°C for 30 min followed by immunoblotting. Antibodies and working concentrations for immunoblotting are indicated in the (**Supplementary Table 2)**. anti-PDE1C (Fabgenix) was used for immunoblotting to determine the knockdown efficiency of PDE1C following 48 h siRNA treatment and anti-PDE1C (Santa Cruz) was used for immunoblotting of PDE1C following immunoprecipitation of PDE1C.

RNA isolation, reverse transcription and qPCR

HASMC RNA was isolated using the Qiagen RNeasy (Qiagen) mini kit as per manufacturer's instructions followed by measurement of RNA purity and concentration using a Nanodrop 1000 (Thermo Scientific). cDNA was synthesized using a Qiagen Omniscript RT according to the manufacturer's instructions. qPCR reactions were performed using PowerUP™ SYBR™ Green Master Mix (Thermo Fisher Scientific) with 2 ng cDNA template and the following primers used are indicated in (**Supplementary Table 3)**. Thermocycler conditions were the following using the QuantStudio 5 Real-Time PCR System: PCR Stage: Step 1 95°C 15 min, Step 2 60°C 1 min repeated 40X, Melt Curve Stage: Step 1 95°C 15 min, Step 2 60°C 1 min, Step 3 Dissociation 95°C 1s.

Puncta visualization and quantification of STIM1 to ORAI translocation

HASMCs were transfected with myc-ORAI1 and GFP-STIM1 using 2µg of DNA using the HASMC nucleofector kit (Lonza) according to the manufacturer's instructions and the Nucleofector™ 2b Device (Lonza). Following 24 h post DNA transfection, cells were plated on gelatin-coated coverslips (ddH_2O supplemented with 0.25% gelatin) and following 48 h post DNA transfection, cells were subjected to Krebs buffer for 5 min, followed by store depletion (Ca^{2+} free + CPA 10 µM) for 5 min. After treatment with Krebs, or store depletion, cells were fixed using paraformaldehyde (4% (vol/vol)), rinsed with HBSS and incubated for 1 h at room temperature with the following primary antibodies: anti-c-myc mouse monoclonal (Sigma 1:1000) and anti-GFP monoclonal rabbit (Santa Cruz 1:1000). The coverslips were then washed with HBSS and incubated with fluorescently-labelled Alexa-conjugated (488 nm) secondary antibodies and with phalloidin-tetramethylrhodamine B isothiocyanate (1:1000) and DAPI (1:1000) (0.3% BSA diluted in HBSS). Imaging was performed using the Leica TCS SP8 confocal microscope (Leica). White light laser (WLL) system was adjusted with settings for the laser line 499 (excitation of Alexa-fluor 488; GFP-STIM1) and 572 nm (excitation for Alexa-fluor 568; myc-Orai1); and UV laser (405 nm) to identify DAPI nucleolus. Individual cells were imaged through a z-stack (Z-dimension of 2-3 µm; pixel size of 0.29 µm) acquisition mode using the HC PL APO CS2 63x/1.40 oil objective. The analysis was performed with the LAS-X Software (Leica Microsystems). To measure the relative mobilization distance of GFP-STIM1 to myc-Orai1 (cell surface), three straight regions of interest (ROI) were drawn per cell initiating it at the central point of each nucleolus and extending to the last bright point representing the cell surface. The intensity of each marker (DAPI, GFP-STIM1 and myc-Orai1) was measured through the ROI (see **Supplementary Fig. 5**). Any intensity peak lower than 5,000 (AU) was considered baseline background. Length measurements of the last peak (ρ) of DAP (ρDAPI), GFP-STIM1 (ρSTIM1) and myc-Orai1 (ρOrai1) were taken, and the following formula was applied to obtain the relative STIM1 distance to Orai1. The relative STIM1 distance (RdSTIM1) equals 1 was suggestive of STIM1 trafficking to the cell surface.

Statistical Analysis

All data presented were analyzed using GraphPad Prism Software and used for statistical analysis. Data in this study was collected from at least three independent experiments unless otherwise stated and presented as means ± SE. A p value <0.05 was considered significant.

Supplementary Figure Legends

Supplementary Figure 1. mTurq2ΔEPACcp173Ven_Ven FRET sensor is sensitive to ADCY inhibition and activation.
A, representative single-cell traces measuring changes in cAMP where decreases in the FRET emission (YFP/CFP) indicate an increase in cAMP signal, using the mTurq2ΔEPACcp173Ven_Ven FRET sensor in control (black trace) and SQ 22536 (1 mM) (blue trace) pretreated HASMCs subjected to an increasing concentration of the ADCY activator forskolin, to confirm that the probe is sensitive to ADCY inhibition and activation by SQ 225356 and forskolin respectively. *B,* measurements in the peak FRET response of cells treated with increasing concentrations of forskolin (+/- SQ 22536) relative to the total FRET response of each individual cell. Three groups of comparisons were conducted using a two-way ANOVA, (between control (black) and SQ 22536 (blue) pretreated cells, between increasing forskolin concentration in control cells, and between increasing forskolin concentration in SQ 22535 pretreated cells) in n=10 control cells and n=9 SQ 22536 pretreated cells, F (3, 68) = 1.247, p=0.2996 interaction, F (3, 68) = 18.74, p<0.0001 (within different Fsk concentrations), F (1, 68) = 206.8, p<0.0001 (control versus SQ treatment). Tukey's multiple comparison ****p<0.0001 (control versus SQ 22536 pretreated cells), #p=0.0172, ###p=0.0004, ####p<0.0001 (control Fsk 0.01 μM versus 0.1 μM, 1 μM and 10 μM Fsk treated cells respectively), p=0.0134 (Fsk 0.01 μM (+SQ 22356) versus Fsk 10 μM (+SQ 22356). *C,* pseudocoloured images of HASMCs indicating changes in FRET subjected to increasing concentrations of the ADCY activator forskolin in control and SQ 22536 pretreated HASMCs. Arrows indicate the addition of forskolin at the concentration indicated. Cooler colours indicate lower (YFP/CFP) ratios and higher cAMP levels, scale bars, 20 μm.

Supplementary Figure 2. Changes in HASMC PKA activity associated with ER(Ca^{2+}) store depletion or SOCE activation. *A,* model of the PKA activity sensor, AKAR4, in which a PKA-substrate domain (LRRATLVD) is positioned between a cerulean (donor, blue) and a Venus (acceptor, yellow) domain (top), and representing how increases in phosphorylation of the PKA-substrate domain decreases the distance between the donor and acceptor domains, thus increasing FRET (bottom). *B,C, r*epresentative pseudo-coloured images of individual HASMCs (scale bar 10 μm) under initial experimental conditions (Ca^{2+}-free Krebs buffer, "0 Ca^{2+}"), during ER(Ca^{2+}) store depletion (Ca^{2+}-free Krebs buffer supplemented with CPA (10 μM), "0 Ca^{2+} + CPA"), during SOCE (Krebs buffer supplemented with CPA (10 μM), 2mM Ca^{2+} + CPA") and, lastly, during sensor saturation (Forskolin (10 μM) + IBMX (100 μM) and representative trace of normalized FRET emission ratio measured in AKAR4-expressing HASMCs under these conditions, respectively. *D, E,* maximal increase in FRET/PKA activity during ER(Ca^{2+}) store depletion (D) or (E) during SOCE, relative to preceding baseline, respectively (n=21 cells, Student's paired t-test****p<0.0001).

Supplementary Figure 3. Characterization of compound 33 (C33).
The molecular structure of compound 33 (C33) and the IC_{50} values in (nM) for PDE1, PDE2, PDE3, PDE4 and PDE5 families.

Supplementary Figure 4. Contribution of Ca^{2+} regulation by PDEs expressed in HASMCs.
A, representative single-cell traces measuring changes in fura-2 fluorescence ratio (R 340/380) in HASMCs using siRNA targeting PDE5A, PDE3A and PDE4D subjected to the SOCE protocol:

preincubation of HASMCs with Ca^{2+} containing Krebs buffer, 5min incubation with Ca^{2+} free Krebs + CPA (10 µM) to deplete ER (Ca^{2+}), 10 min incubation with Ca^{2+} containing Krebs buffer + CPA (10 µM) to induce SOCE to replenish ER (Ca^{2+}). *B,* quantification in the peak change (R 340/380) during SOCE (Ca^{2+} containing Krebs buffer + CPA (10 µM)) and normalized to control siRNA, n=48-56 cells, one-way ANOVA, F=5.386, p=0.0014, followed by Dunnett's multiple comparisons **p=0.0014. *C,* quantification in the peak change (R 340/380) during SOCE (Ca^{2+} containing Krebs buffer + CPA (10 µM)) in HASMCs treated with the PDE5 inhibitor sildenafil (100 nM), PDE3 inhibitor cilostamide (5 µM) and PDE4 inhibitor rolipram (10 µM). Data normalized to the control, n=33-58 cells, using a one-way ANOVA, F=6.016, p=0.0006, followed by Dunnett's multiple comparisons, *p=0.0173.

Supplementary Figure 5. PDE1C silencing does not alter STIM1 translocation to the membrane following store depletion or alter STIM1 protein expression. *A,* representative low and high magnification of high-resolution deconvoluted confocal images of HASMCs co-transfected with GFP-STIM1 and myc-Orai1 and incubated in Krebs (2 mM Ca^{2+}) for 5 min or in Ca^{2+} free Krebs supplemented with (10 µM) CPA to deplete ER (Ca^{2+}). Inset indicates region of interest zoomed in. Cells visualized by staining with 488-conjugated secondary for GFP-STIM1, 568-conjugated secondary for myc-Orai1 and with DAPI; scale bars, 20 µm. *B,* relative distance of STIM1 to Orai1 quantified and normalized to control Krebs. Unpaired Student's t-test, n=5-7 cells in each group, *p=0.0473. *C,* representative immunoblot of STIM1 expression using the rabbit polyclonal STIM1 antibody following PDE1C silencing. n=3, unpaired Student's t-test. *D,* quantification of the relative mobilization distance of STIM1 to Orai1 (cell surface) was conducted by drawing three straight regions of interest (ROI) from the central point of each nucleolus and extending the lines to the last bright point representing the cell surface; scale bar, 20 µm. For clarity of presentation, the same image as shown in A was used to demonstrate how distances were calculated in D. The intensity of each marker (DAPI, STIM1 and Orai1) was measured through the ROI (blue trace represents DAPI intensity, red trace represents Orai1 intensity and green trace represents STIM1 intensity). Any intensity peak lower than 5,000 (Arbitrary Units) was considered baseline background. Length measurements of the last peak (ρ) of DAPI (ρDAPI), STIM1 (ρSTIM1) and Orai1 (ρOrai1) were taken, and the indicated formula was applied to obtain the relative STIM1 distance to Orai1.

Supplementary Figure 6. PDE4 does not regulate cAMP mediated changes during SOCE in HASMCs.

A, representative single-cell traces measuring changes in cAMP using the mTurq2ΔEPACcp173Ven_Ven FRET sensor in control and Ro 20-1724 (10 µM) treated HASMCs subjected to the SOCE protocol: preincubation with Ca^{2+} free containing Krebs buffer, followed by 5min incubation with Ca^{2+} free containing Krebs buffer + CPA (10 µM) to deplete ER (Ca^{2+}), followed by 10min incubation with Ca^{2+} containing Krebs buffer + CPA (10 µM). *B,* quantification in the percent decrease in FRET (YFP/CFP) (ie. increase in cAMP) during ER (Ca^{2+}) depletion; Ca^{2+} free + CPA (10 µM). *C,* quantification in the percent increase in FRET (YFP/CFP) (ie. decrease in cAMP) during the initial transient phase in SOCE; Ca^{2+} + CPA (10 µM). *D,* quantification in the percent decrease in FRET (YFP/CFP) (ie. increase in cAMP) during the second phase of SOCE; Ca^{2+} + CPA (10 µM). Analysis was conducted in control n= 21 and Ro 20-1724 n=19 cells, Student's unpaired t-test.

Supplementary Figure 7. PDE1C silencing promotes leading edge structures in HASMCs.
A, schematic of the leading edge transwell assay. HASMCs are plated on top of a porous 3 μm Fluoroblok^{TM} transwell filter and allowed to form leading edge protrusions for 4 h in response to a chemotactic factor (FBS 0.5%). *B,* representative images of the underside of Fluoroblok^{TM} transwells of chemotactic HASMCs subjected to the leading-edge assay in cells transfected with siRNA control or siRNA PDE1C using an alternative siRNA for PDE1C (PDE1C #2). TRITC-conjugated phalloidin is used to visualize F-actin, scale bars 50 μm. *C,* quantification of LEPs migrated to the bottom of the transwell by normalizing data to siRNA control, from n=3 experiments, Student's unpaired t-test, ***p<0.0001. *D,* knockdown efficiency of mRNA PDE1C is measured following 48 h of transfecting HASMCs with the alternative siRNA targeting PDE1C (PDE1C # 2) and normalized to the negative siRNA control from n=3 experiments, Student's unpaired t-test, **p=0.0037.

Supplementary Figure 8. SOCE is greater at the leading edge of migrating HASMCs compared to the cell body. *A,* representative single-cell fura-2 traces measuring Ca^{2+} in HASMCs at the cell body (soma) or at the front (leading edge) during post ER(Ca^{2+}) depletion SOCE activation. *B,C,* comparing (R340/380) at the soma *versus* the leading edge in migrating HASMCs at baseline, or during SOCE activation. n=9 cells, *p=0.0144, Student's paired t-test.

Supplementary Figure 9. Graphical Abstract. *A* depicting relative ER(Ca^{2+}) concentration in polarized migrating cell. *B* scheme of steps involved in PDE1C-mediated regulation of SOCE and of SOCE-mediated activation of ADCY8.

Supplementary Figure 1

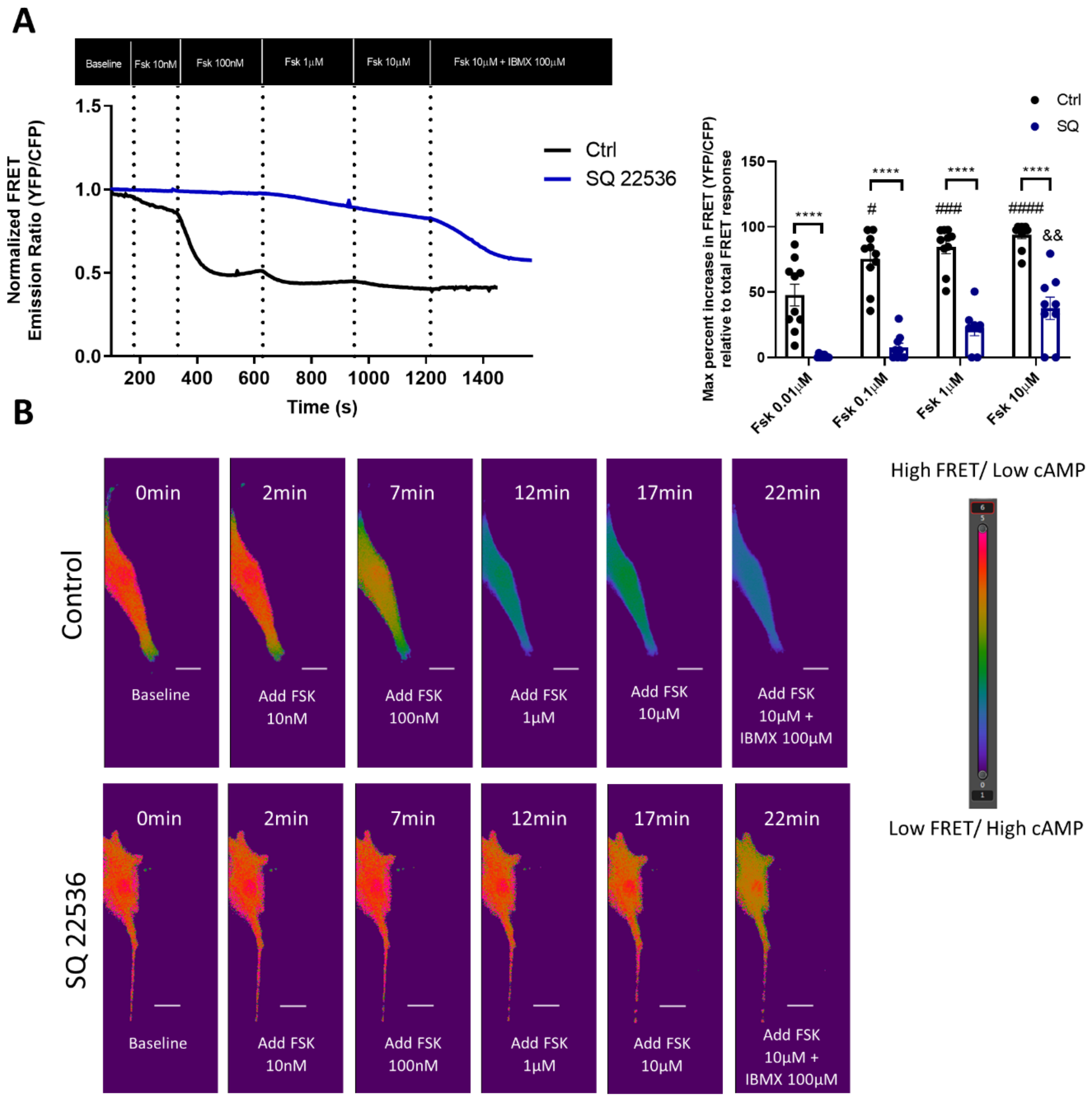

Supplementary Figure 2

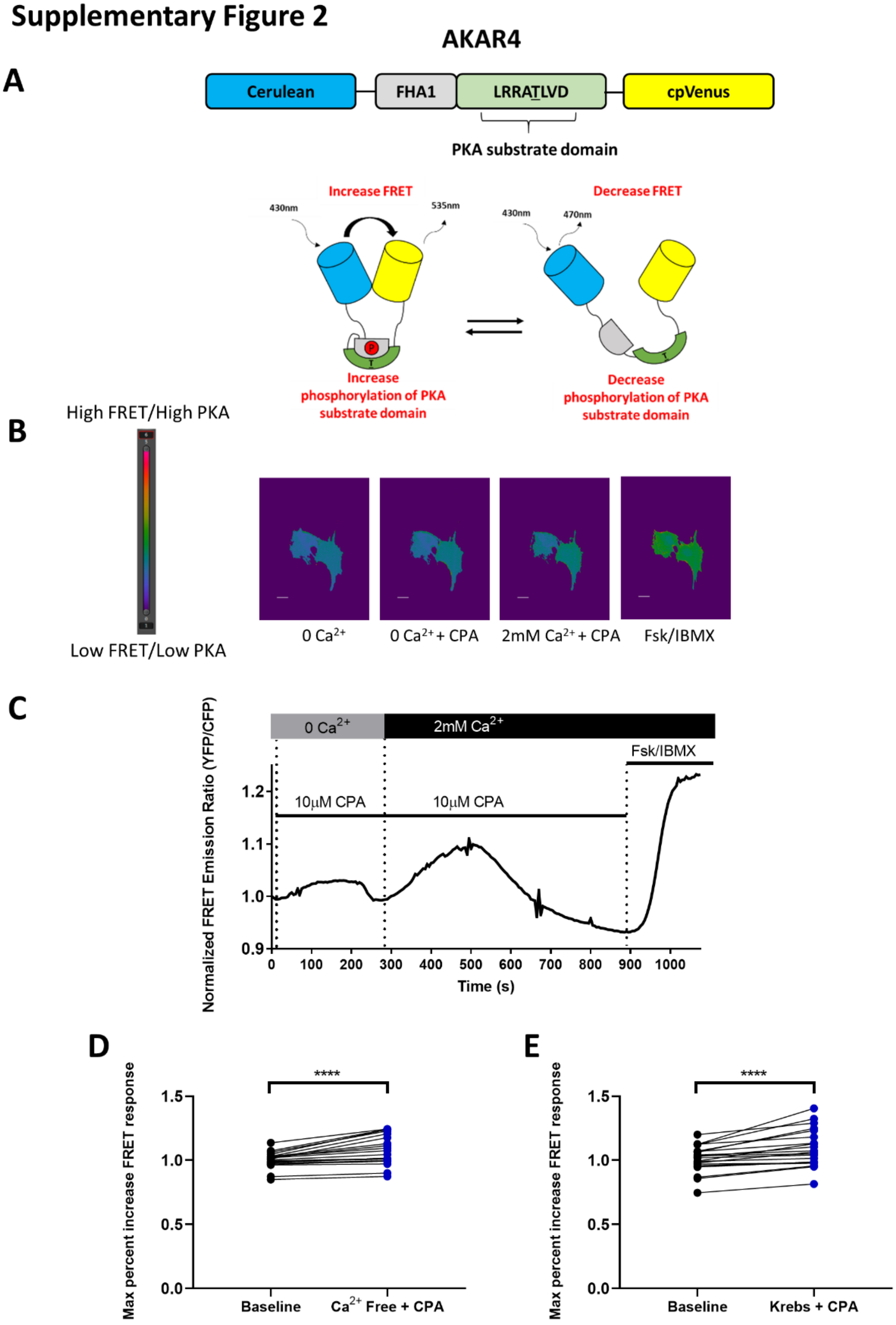

Supplementary Figure 3

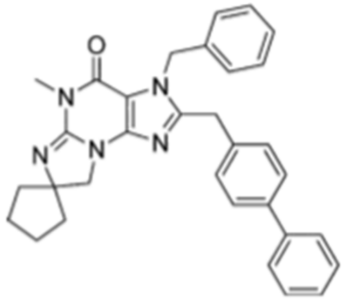

C33 IC_{50} (nM)				
PDE1	PDE2	PDE3	PDE4	PDE5
0.6	1800	7000	200	200

Supplementary Figure 4

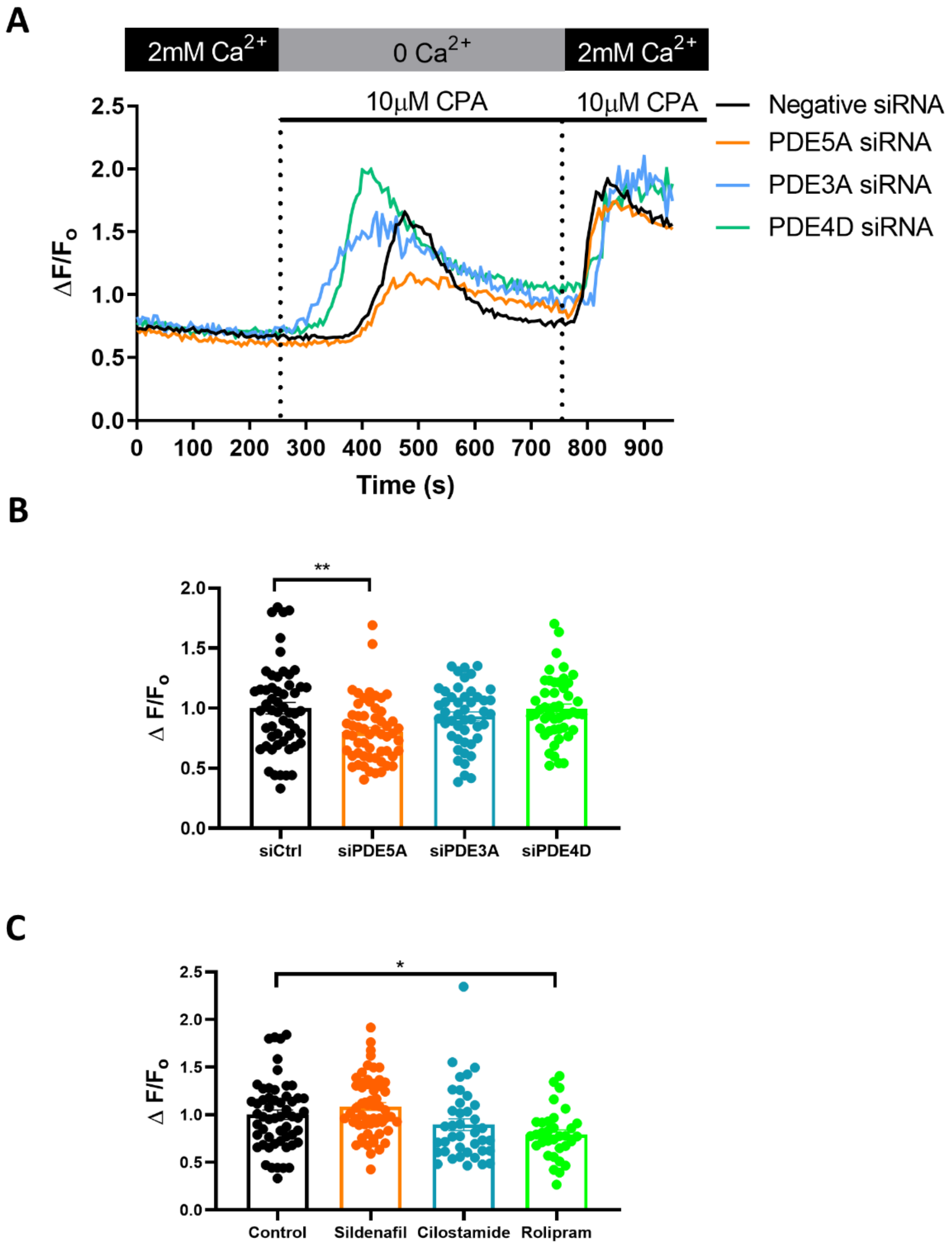

Supplementary Figure 5

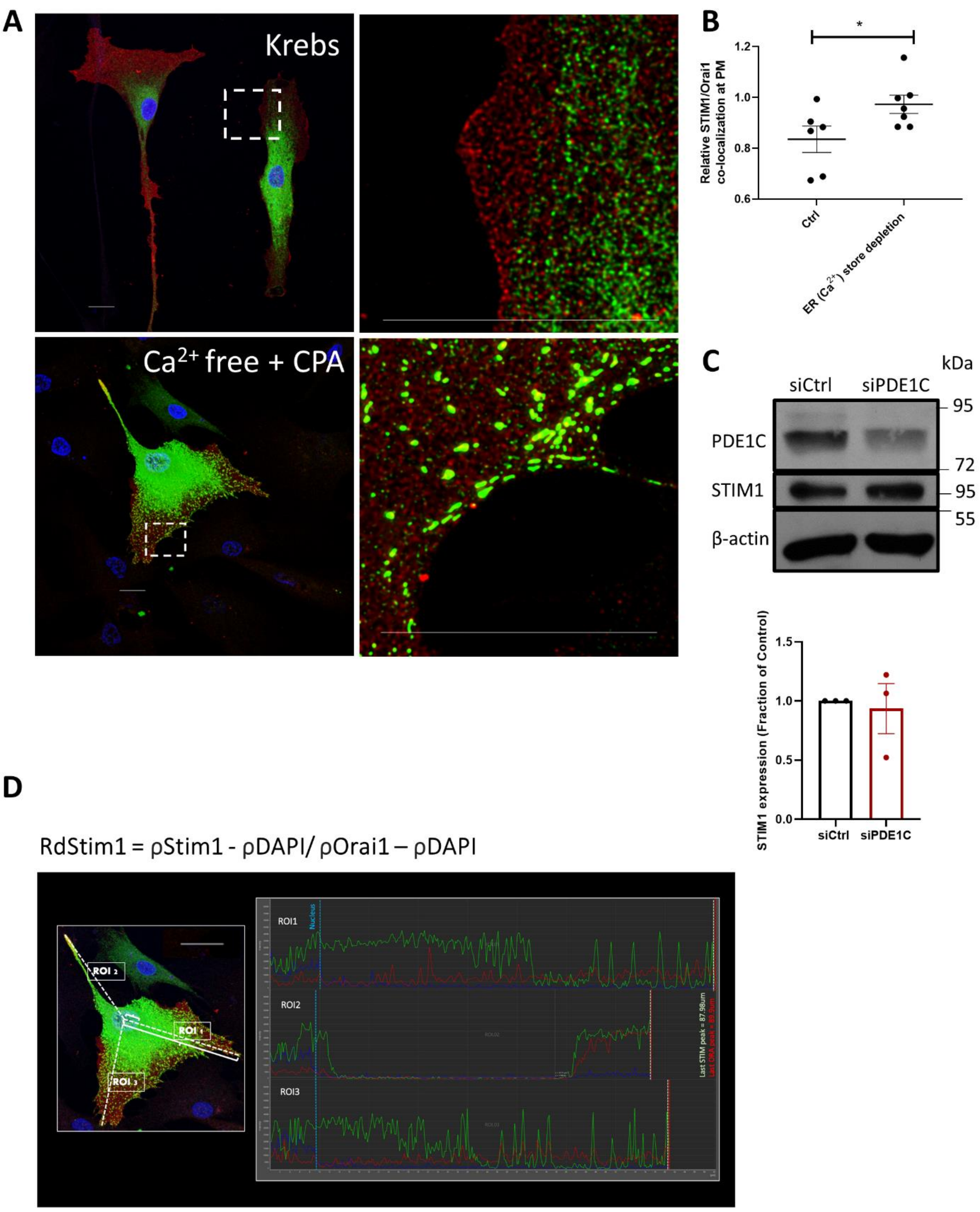

Supplementary Figure 6

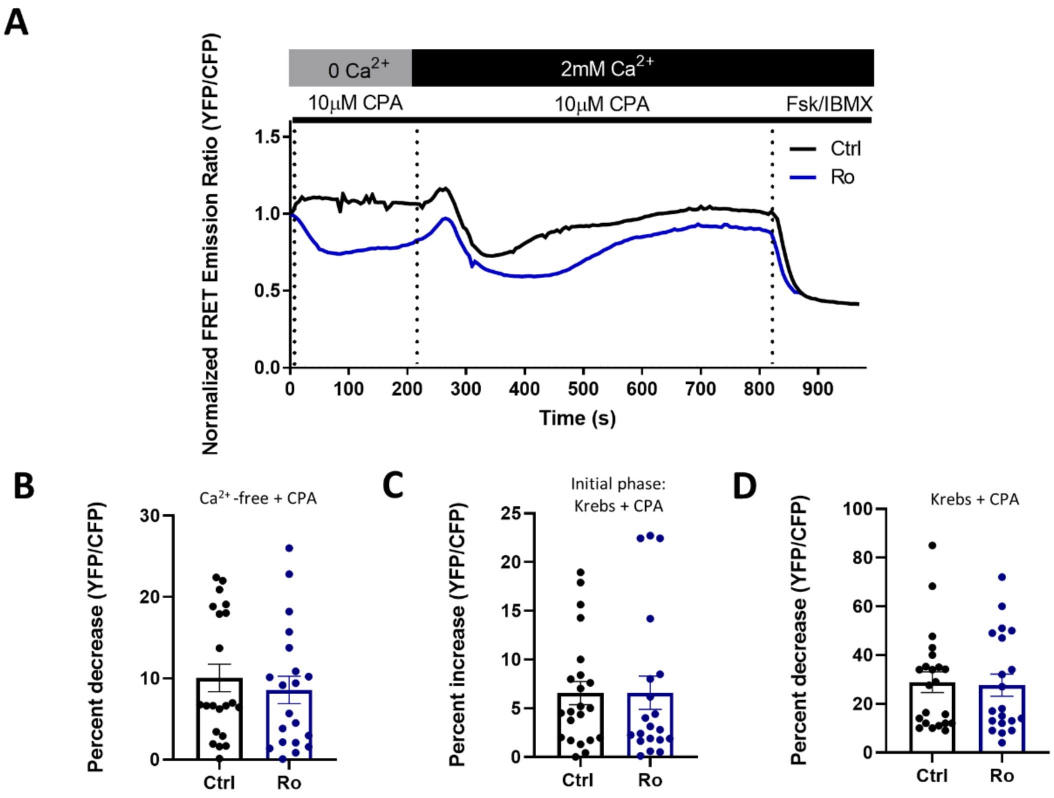

Supplementary Figure 7

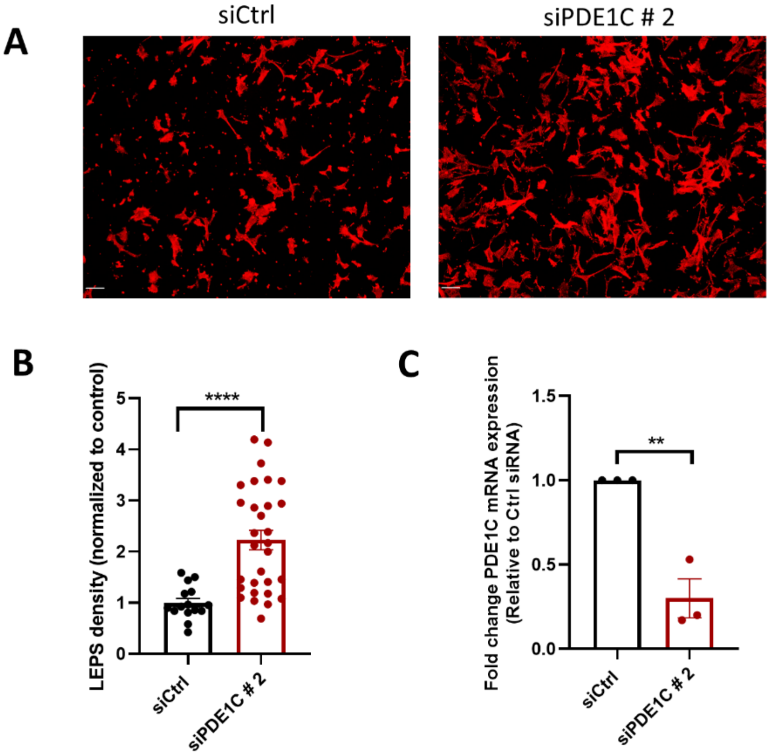

Supplementary Figure 8

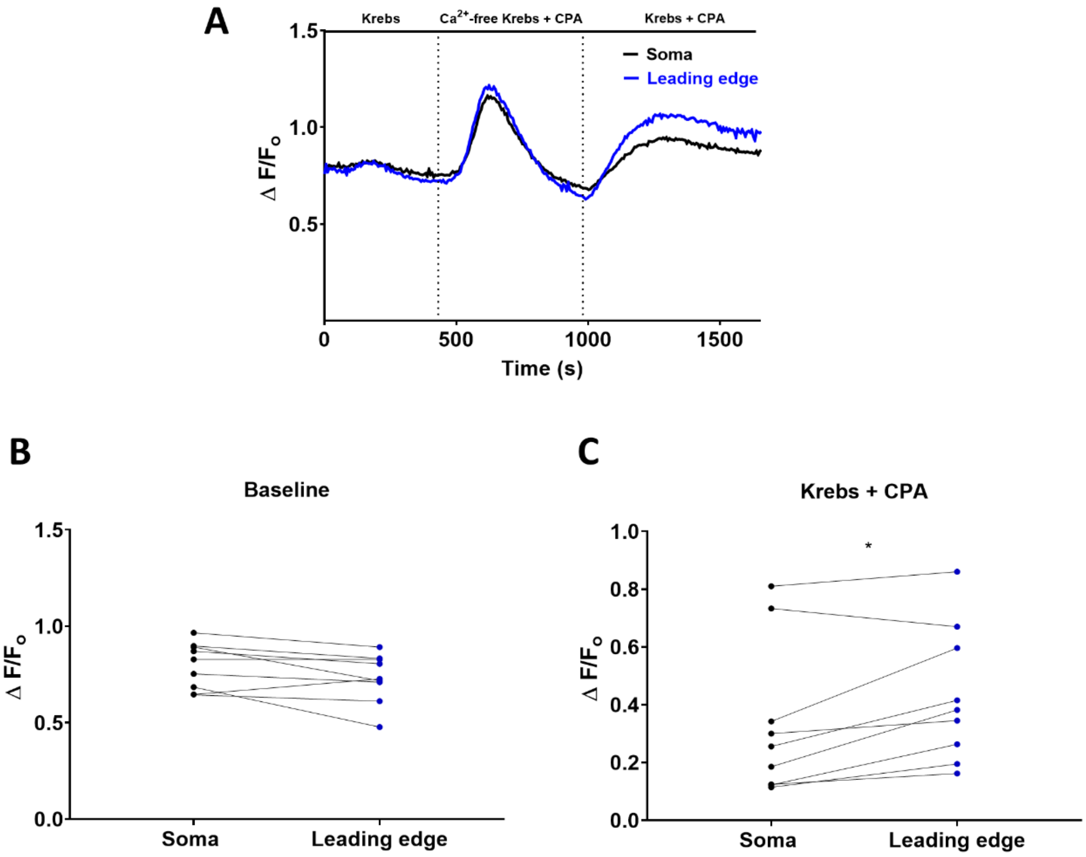

Reciprocal Regulation of SOCE and cAMP Signaling and Human Arterial Smooth Muscle Cell Migration

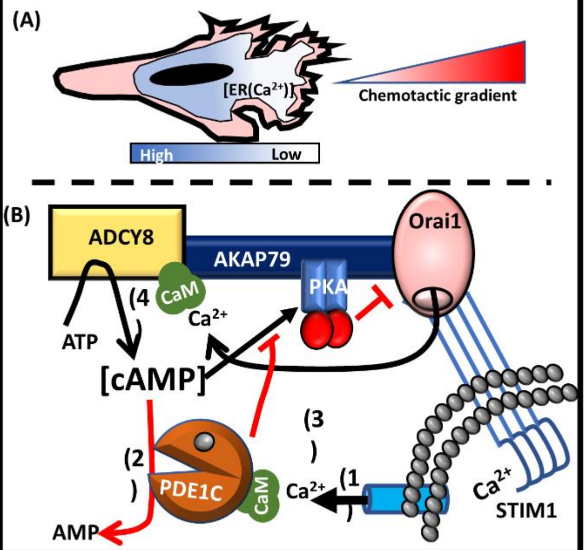

A) A gradient of ER(Ca^{2+}) exists in polarized, motile, cells.

B) ER(Ca^{2+}) depletion (1), increases PDE1C cAMP hydrolysis (2), reducing Orai1 inactivation (3), increasing SOCE-mediated ADCY8 activation.

Supplemental Table 4. PDE1C cAMP PDE activity in HASMCs 48 h post-siRNA treatment.

RNAi	Ca^{2+}/CaM stimulated cAMP PDE activity (pmol/min/mg)	cAMP PDE Activity (% of Basal PDE activity)	
		PDE3	PDE4
Control	57.7 ± 17.6	43.5 ± 18.8	64.9 ± 11.0
PDE1C	14.6 ± 13.2	46.2 ± 7.3	57.2 ± 3.5

Ca^{2+}/CaM stimulated cAMP PDE activity in HASMCs transfected with control or PDE1C siRNA and representative data of n=3 independent experiments, $p<0.05$ comparing Ca^{2+}/CaM stimulated cAMP PDE activity in control versus PDE1C siRNA transfected HASMCs. % cAMP PDE activity of HASMC PDE3 and PDE4 in HASMCs treated with control or PDE1C siRNA and representative data of n=2 independent experiments is presented as a percentage of total basal HASMC cAMP PDE activity in means ± SD, p= 0.8673; comparing cAMP PDE3 activity in control versus PDE1C siRNA transfected HASMCs, p=0.4451; comparing cAMP PDE4 activity in control versus PDE1C siRNA transfected HASMCs.
